# Ubiquitin-based pathway acts inside chloroplasts to regulate photosynthesis

**DOI:** 10.1126/sciadv.abq7352

**Published:** 2022-11-16

**Authors:** Yi Sun, Zujie Yao, Yiting Ye, Jun Fang, Honglin Chen, Yuping Lyu, William Broad, Marjorie Fournier, Genyun Chen, Yonghong Hu, Shabaz Mohammed, Qihua Ling, R. Paul Jarvis

**Affiliations:** ^1^ Department of Plant Sciences, University of Oxford, Oxford OX1 3RB, UK.; ^2^ National Key Laboratory of Plant Molecular Genetics, CAS Centre for Excellence in Molecular Plant Sciences, Institute of Plant Physiology and Ecology, Chinese Academy of Sciences, Shanghai, China.; ^3^ University of Chinese Academy of Sciences, Beijing, China.; ^4^ Department of Biochemistry, University of Oxford, Oxford OX1 3QU, UK.; ^5^ Shanghai Chenshan Plant Science Research Center, Chinese Academy of Sciences, Shanghai, China.; ^6^ Advanced Proteomics Facility, University of Oxford, Oxford OX1 3QU, UK.; ^7^ Department of Chemistry, University of Oxford, Oxford OX1 3TA, UK.; ^8^ Rosalind Franklin Institute, Oxfordshire OX11 0FA, UK.; ^9^ CAS-JIC Center of Excellence for Plant and Microbial Sciences (CEPAMS), Institute of Plant Physiology and Ecology, Chinese Academy of Sciences, Shanghai, China.

## Abstract

Photosynthesis is the energetic basis for most life on Earth, and in plants it operates inside double membrane–bound organelles called chloroplasts. The photosynthetic apparatus comprises numerous proteins encoded by the nuclear and organellar genomes. Maintenance of this apparatus requires the action of internal chloroplast proteases, but a role for the nucleocytosolic ubiquitin-proteasome system (UPS) was not expected, owing to the barrier presented by the double-membrane envelope. Here, we show that photosynthesis proteins (including those encoded internally by chloroplast genes) are ubiquitinated and processed via the CHLORAD pathway: They are degraded by the 26*S* proteasome following CDC48-dependent retrotranslocation to the cytosol. This demonstrates that the reach of the UPS extends to the interior of endosymbiotically derived chloroplasts, where it acts to regulate photosynthesis, arguably the most fundamental process of life.

## Teaser

The ubiquitin-proteasome system targets proteins inside chloroplasts, including chloroplast-encoded photosynthesis subunits.

## INTRODUCTION

Chloroplasts are the defining organelles of plants and algae, and evolved through endosymbiosis from a cyanobacterial ancestor (*1*). They use light energy to convert CO_2_ into carbohydrate through photosynthesis and thus play a pivotal role in controlling atmospheric CO_2_ levels. Apart from photosynthesis, chloroplasts have a multiplicity of metabolic functions [including the synthesis of amino acids, fatty acids (FAs), and plant hormones] and have a correspondingly diverse proteome (*2*). Homeostatic mechanisms governing the chloroplast proteome, including proteolysis, are consequently of vital importance for organellar functions and plant development.

The chloroplast proteome comprises approximately 3000 proteins, >90% of which are encoded in the nucleus with the remainder encoded by the chloroplast genome (*1*, *2*). Several classes of internal protease of prokaryotic origin participate in chloroplast proteostasis, and many of these contribute to the regulation of photosynthesis (*3*, *4*). Photosynthesis is a highly complex process carried out by distinct functional units, each one with numerous protein components: photosystems I and II (PSI and PSII), linked by the cytochrome b_6_f complex, for light harvesting; adenosine 5′-triphosphate (ATP) synthase; and the Calvin cycle for carbon fixation. Proteolytic regulation of the D1 subunit of the PSII reaction center by internal FtsH and Deg proteases, which is crucial for maintaining photosynthetic efficiency, has been studied intensively (*5*, *6*). However, mechanisms underlying the proteolysis of many other photosynthesis components remain unclear.

Our recent work uncovered a role for the ubiquitin-proteasome system (UPS) in the regulation of chloroplast outer envelope membrane (OEM) proteins, via a pathway termed CHLORAD (chloroplast-associated protein degradation) (*7*, *8*). CHLORAD involves the RING (really interesting new gene)–type ubiquitin E3 ligase SP1 (suppressor of *ppi1* locus 1), the Omp85-type β-barrel channel SP2 (suppressor of *ppi1* locus 2), and the AAA^+^ (adenosine triphosphatase associated with diverse cellular activities) chaperone CDC48. The SP1 and SP2 proteins form a complex in the OEM, with SP1 mediating ubiquitination of target proteins and SP2 forming a retrotranslocon that delivers ubiquitinated targets to the cytosol, for degradation by the 26*S* proteasome. The motive force for this retrotranslocation is provided by CDC48. Known targets of CHLORAD are the TOC (translocon at the outer envelope membrane of chloroplasts) proteins that mediate chloroplast protein import (*9*–*12*), the regulation of which allows CHLORAD to control organellar development and functions, for example, in response to stress conditions (*13*).

In contrast with the functionally analogous ERAD (endoplasmic reticulum-associated protein degradation) system, which eliminates a broad range of both misfolded, dysfunctional and normal, functional endoplasmic reticulum (ER) proteins (*14*), the data published to date suggest that CHLORAD has a narrower focus on the protein import machinery in the chloroplast OEM. Given that CHLORAD is heavily involved in the profound structural and functional changes that chloroplasts (and other plastid types) undergo during plant development (*7*, *8*, *12*, *15*), it is conceivable that CHLORAD has a broader role that includes, for example, the removal of those proteins that become redundant during such dynamic processes. As chloroplasts have a diversity of functions (*1*, *2*), it is possible that many as yet unknown substrates of CHLORAD exist in chloroplasts, particularly in the interior, which accounts for more than 99% of total chloroplast protein (*16*).

## RESULTS

### Detection of internal chloroplast ubiquitination by fractionation and affinity purification

**Fig. 1.**
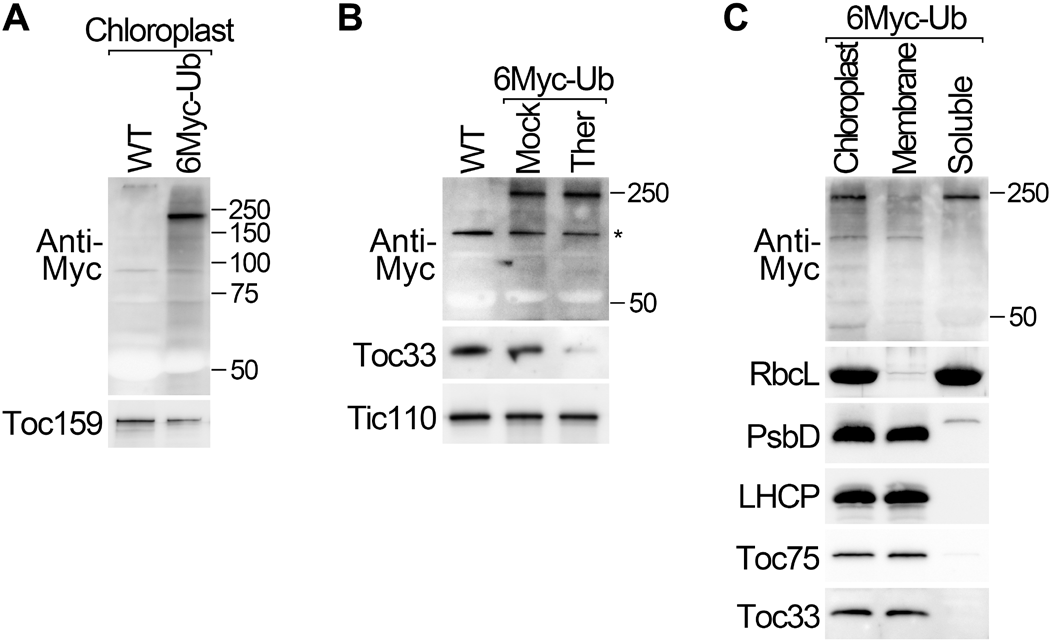
Proteins of the chloroplast interior are polyubiquitinated. (**A** and **B**) Chloroplasts isolated from transgenic plants expressing Myc-tagged ubiquitin (6Myc-Ub), and from wild type, were analyzed by immunoblotting (A). Similar chloroplasts were treated with thermolysin protease (Ther), or buffer lacking protease (Mock), before immunoblotting analysis (B). The asterisk in (B) indicates a nonspecific band. (**C**) Chloroplasts isolated from 6Myc-Ub plants were separated into membrane pellet and soluble supernatant (predominantly stroma) fractions by centrifugation at 18,000*g* and then analyzed by immunoblotting. Analysis of control proteins confirmed the efficacy of the protease treatment and fractionation steps. Positions of molecular weight markers (sizes in kDa) are shown to the right of the images.

To gain initial insight into the possibility that CHLORAD is wider in scope than previously envisaged, we conducted an immunoblot investigation of isolated *Arabidopsis* chloroplasts. To improve the sensitivity of this analysis, a transgenic plant line expressing 6× Myc-tagged ubiquitin (6Myc-Ub) was used. We detected high–molecular weight smears in purified chloroplasts upon anti-Myc analysis (Fig. 1A). Moreover, these anti-Myc smears persisted following treatment of the chloroplasts with thermolysin (a protease that removes surface-exposed OEM proteins; Fig. 1B) (*17*), indicating that ubiquitinated proteins may exist in internal compartments, such as the inner envelope membrane (IEM), stroma, or thylakoid membranes. Support for the existence of ubiquitinated proteins in the stroma (the main aqueous compartment of the organelle) was provided by subfractionation, which revealed the presence of polyubiquitin smears in both membrane and soluble (predominantly stroma) fractions (Fig. 1C).

To verify that the observed smears correspond to ubiquitinated chloroplast proteins, they were enriched by anti-Myc immunoprecipitation and analyzed by mass spectrometry. A number of internal chloroplast proteins were identified, and for one of them, a specific ubiquitination site was detected: This was the stromal protein PrfB3 (peptide chain release factor 3), which functions in chloroplast gene expression (fig. S1 and table S1) (*18*). Thus, together, these data supported the hypothesis that CHLORAD acts on proteins inside chloroplasts.

### Ubiquitinomics reveals that proteins involved in photosynthesis are ubiquitinated

To develop a more complete picture of the substrates of CHLORAD, we sought to systematically identify ubiquitination targets in chloroplasts and their sites of modification. First, chloroplasts purified from wild-type plants were analyzed using a ubiquitin remnant (di-Gly) antibody-based peptide enrichment ubiquitinomic approach (*19*), and the samples were subjected to proteomic analysis. In total, 57 nonredundant ubiquitination sites in 40 proteins were identified (table S2). To increase the sensitivity of detection, retrotranslocation of CHLORAD substrates was blocked using a dominant-negative mutant of CDC48 (CDC48-DN) (*8*); a much stronger ubiquitination signal could be detected in isolated chloroplasts following CDC48-DN induction (fig. S2A). Chloroplasts purified from CDC48-DN plants were analyzed using the same ubiquitinomic approach (*19*), and three independent experiments were performed. The ubiquitinated proteins were identified by cross-referencing with a custom-assembled chloroplast proteome database comprising 4174 proteins (*16*, *20*, *21*). In total, 768 nonredundant ubiquitination sites in 316 proteins were identified in at least one experiment for CDC48-DN (table S3 and fig. S2). These results indicated that the chloroplast proteome is broadly ubiquitinated and that ubiquitinated chloroplast proteins are commonly processed by CDC48.

**Fig. 2.**
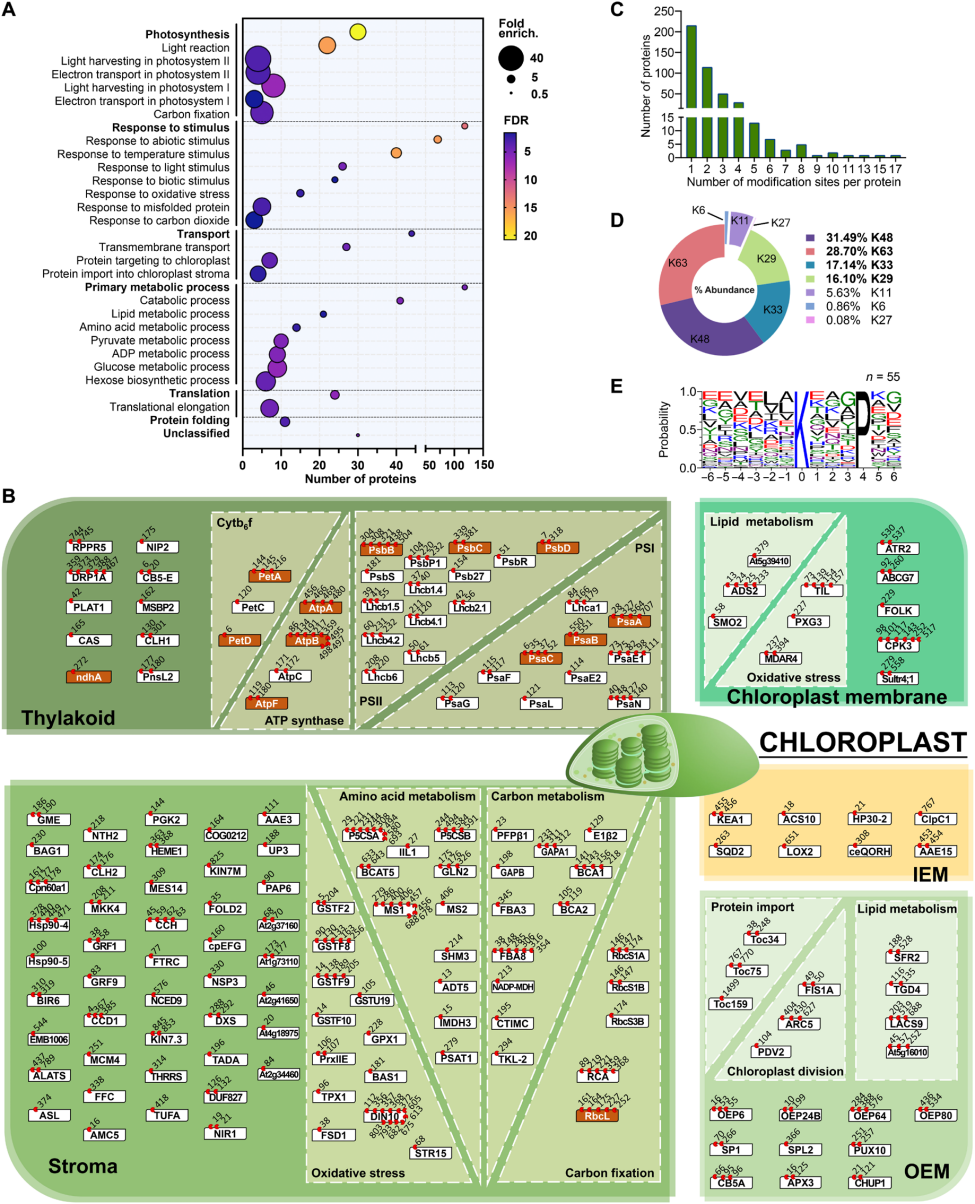
Photosynthesis and other proteins of the chloroplast interior are prominent in the chloroplast ubiquitinome. (**A**) Dot plot showing significantly overrepresented gene ontology (GO) terms in the chloroplast ubiquitinome, as determined using chloroplasts purified from CDC48-DN plants after estradiol induction. Dot size indicates overrepresentation (fold enrichment) compared to the whole genome. Dot color indicates false discovery rate [FDR; −log_10_ (*P* value)], where higher FDR values indicate more statistically significant enrichment. Dots are not shown for terms lacking statistically significant (*P* < 0.05) enrichment. (**B**) Suborganellar and functional distribution of the chloroplast ubiquitinome, showing also ubiquitination sites as determined by di-Gly analysis. Localizations were assigned manually, and only proteins localized in a defined chloroplast compartment (OEM, IEM, stroma, and thylakoid), or in an internal chloroplast membrane fraction (comprising IEM and thylakoids), are shown. Boxes indicate individual proteins (white, nucleus-encoded; orange, chloroplast-encoded), and red circles with numbers indicate which amino acids showed ubiquitination. (**C**) Histogram showing the number of ubiquitination sites detected per protein in the chloroplast ubiquitinome. (**D**) Pie chart showing the relative abundance (based on peptide intensity) of different polyubiquitin linkage types in the chloroplast ubiquitinome. Values are means from three experiments. (**E**) Logo plot showing motif analysis of ubiquitinated peptides in the chloroplast ubiquitinome. Six residues either side of the modification site (position 0) are shown.

The identified putative CHLORAD substrates belonged to various functional categories but with a noteworthy enrichment in photosynthesis components (Fig. 2A). With regard to suborganellar localization, OEM proteins were well represented in the set (20 of 316 proteins), which is consistent with our published results (*7*, *8*); TOC components (Toc159, Toc75, and Toc34) and SP1 and SPL2 (SP1-Like2) were present as expected, as well as others such as FA synthetase LACS9 (long chain acyl-CoA synthetase 9) and the channel protein OEP24 (outer plastid envelope protein 24) (*16*, *22*). However, a considerable number of IEM, stromal, and thylakoidal proteins were also identified, including the lipoxygenase LOX2 (*23*); ribulose-1,5-bisphosphate carboxylase-oxygenase (RuBisCO) small subunit RbcS; various metabolism-related proteins; light-harvesting chlorophyll-binding proteins (LHCPs); and photosystem subunits such as PsaA, PsaB, and PsbC (Fig. 2B and table S3) (*24*). This suggested that ubiquitination is a major mechanism for the regulation of the chloroplast proteome, particularly regarding photosynthesis.

Among the putative CHLORAD substrates were 13 chloroplast-encoded proteins with identified ubiquitination sites. These included two PSI subunits, four PSII subunits, two cytochrome b_6_f subunits, three ATP synthase subunits, one NDH (NADH dehydrogenase-like) complex subunit, and the RuBisCO large subunit (table S4). This was an important finding because it ruled out the possibility that the detected ubiquitination was the consequence of modification of unimported cytosolic precursors (preproteins) (*25*), although we note that this was already highly unlikely given that the ubiquitinome analysis was conducted using purified chloroplasts.

To verify the results of the ubiquitinome analysis, selected proteins were analyzed using an in vivo ubiquitination assay. Cells expressing FLAG-tagged ubiquitin (FLAG-Ub) were subjected to immunoprecipitation analysis, and the eluates were assessed for the ubiquitination status of putative CHLORAD substrates. This confirmed the ubiquitination of the stromal PrfB3 protein (fig. S3A) (*18*), as well as that of proteins encoded by the chloroplast genome (i.e., PsaA and PsbC; fig. S3B). Moreover, the amount of detectable ubiquitination was enhanced when substrate retrotranslocation was blocked by expressing CDC48-DN (fig. S3B). This provided further strong support for the conclusion that proteins of the chloroplast interior are regulated by ubiquitination and CDC48.

The chloroplast ubiquitinome had 2.7 ubiquitination sites per protein, suggesting extensive ubiquitination of chloroplast proteins (Fig. 2C); the global ubiquitinome has 1.1 sites per protein (*26*). Analysis of the di-Gly data revealed that all polyubiquitin linkage types are present in chloroplasts, with the following distribution, based on integration of signal: Lys^48^ > Lys^63^ > Lys^33^ > Lys^29^ > Lys^11^ >>> Lys^6^/Lys^27^ (Fig. 2D). Because K48 was the most abundant linkage type, much of chloroplast ubiquitinome can be assumed to be primed for proteasomal degradation. We identified a putative consensus motif for ubiquitin attachment (Fig. 2E), which was not observed previously in the global plant ubiquitinome (*26*–*28*), implying that a specific ubiquitination process may occur inside chloroplasts. In addition, ubiquitination sites were identified in different subchloroplastic compartments, although the transmembrane domains clearly lack ubiquitination (fig. S4).

### Quantitative proteomics reveals that CHLORAD regulates the levels of a broad range of proteins

**Fig. 3.**
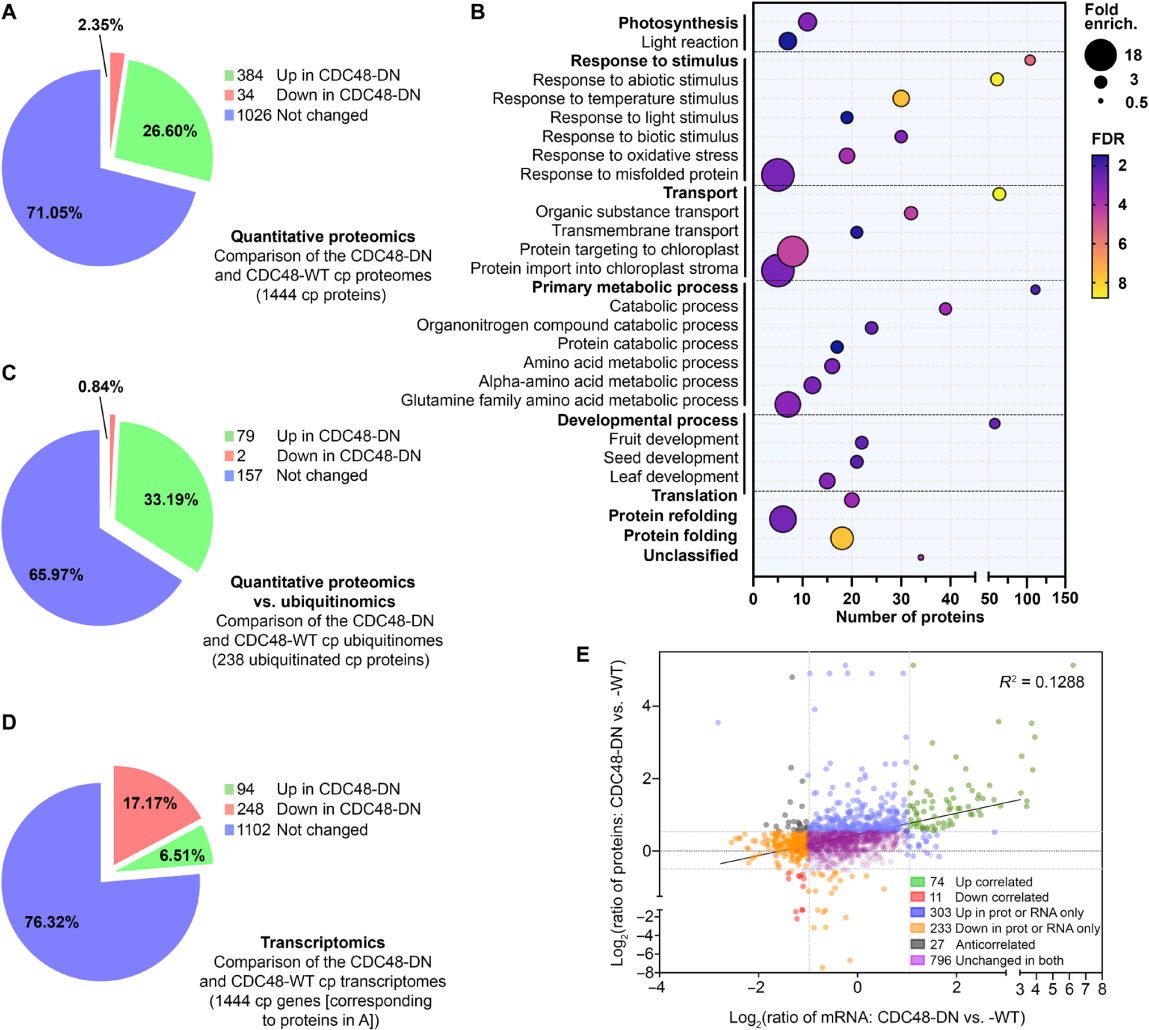
Identification of numerous CHLORAD substrates using quantitative proteomics. (**A**) Pie chart showing the differential accumulation of chloroplast (cp) proteins upon CHLORAD inhibition, as determined by quantitative proteomic analysis of CDC48-DN and CDC48-WT transgenic plants after estradiol induction. (**B**) Dot plot showing significantly overrepresented GO terms for chloroplast proteins that are overaccumulated in CDC48-DN plants. Dot size indicates overrepresentation (fold enrichment) compared to the whole genome. Dot color indicates false discovery rate [FDR; −log_10_ (*P* value)], where higher FDR values indicate more statistically significant enrichment. Dots are not shown for terms lacking statistically significant (*P* < 0.05) enrichment. (**C**) Pie chart showing the differential accumulation of ubiquitinated chloroplast proteins in the quantitative proteomic analysis depicted in (A). Only proteins in the chloroplast ubiquitinome (Fig. 2) are shown. (**D**) Pie chart showing the differential expression of mRNAs corresponding to the chloroplast proteins identified by proteomics in (A), as determined by RNA-seq transcriptomics. (**E**) Dot plot showing a lack of correlation between chloroplast protein abundancies [from (A)] and corresponding mRNA abundancies [from (D)]. The coefficient of determination (*R*^2^) value is shown.

A hallmark of CHLORAD substrates is that their steady-state levels increase upon CHLORAD inhibition (*7*, *8*). Thus, as a complement to the ubiquitinome analysis, we sought chloroplast proteins that overaccumulate after CDC48 inhibition. To this end, we performed label-free quantitative proteomic analysis of plants expressing CDC48-DN or the corresponding CDC48-wild-type (WT) control and then filtered the data using the same custom chloroplast proteome database as used for the ubiquitinomics; this approach avoided the need for a time-consuming chloroplast isolation step that might bias protein quantification. In three biological replicates, we detected 1444 proteins in both genotypes of which ~27% were present at elevated levels (>1.5 fold) in CDC48-DN relative to the control, as expected for CHLORAD targets (Fig. 3, A and B; fig. S5; and table S5); a selection of these data is shown in table S6. The results were highly consistent with our published immunoblot data (*8*): TOC components (Toc159, Toc33, and Toc75) were overaccumulated in CDC48-DN, whereas established nonsubstrate proteins [Tic40 (translocon at the inner envelope membrane of chloroplasts, 40 kDa subunit) and OEP80] were unchanged (table S6). We also conducted label-free quantitative proteomic analysis of chloroplasts isolated from the same CDC48-DN and CDC48-WT plants, and similar results were obtained (table S7).

Among the other elevated proteins in CDC48-DN were a number of OEM proteins unrelated to the core TOC apparatus, including LACS9, OEP64, OEP24 (a channel protein), and CHUP1 (chloroplast unusual positioning 1) (which regulates chloroplast movement), all of which were identified also by ubiquitinomics (*16*, *22*, *29*). Given that the OEM proteome is much less abundant than those of the stroma and thylakoids (*20*), this suggested that OEM proteins are major substrates of CHLORAD, as would be expected. In a further parallel with the ubiquitinome analysis, many IEM and stromal proteins (e.g., LOX2) were also elevated in CDC48-DN. These internal proteins have a range of different functions, including photosynthesis, chlorophyll metabolism, and oxidative stress response.

To verify the proteomics data, immunoblotting and confocal microscopy analyses were performed. Some proteins were analyzed using specific antibodies, and their levels were found to be elevated in CDC48-DN plants, as expected (fig. S6). Where specific antibodies were not available, selected proteins [including LACS9 and FAX1 (fatty acid export 1)] were transiently expressed with a yellow fluorescent protein (YFP) tag and then analyzed by immunoblotting and microscopy (fig. S7). Both analytical approaches showed the proteins to be elevated in CDC48-DN cells, while the latter revealed that the accumulated protein is localized to the chloroplasts in each case (fig. S7). Notably, of the 238 proteins detected in this proteomic analysis that also contain at least one ubiquitination site (tables S3 and S5), 79 (33%) were overaccumulated in CDC48-DN chloroplasts (Fig. 3C), supporting the notion that they are CHLORAD substrates.

Moreover, RNA sequencing (RNA-seq) analysis revealed that, although there are many substantial CDC48-dependent changes in global transcription (fig. S8 and table S8), including that of ubiquitin-related proteolytic genes, most transcripts encoding proteins overaccumulated in CDC48-DN chloroplasts were not up-regulated transcriptionally (Fig. 3, D and E). In fact, mRNAs for chloroplast proteins tended to be reduced in CDC48-DN plants, in contrast to the protein levels (Fig. 3D). In general, we found no correlation between mRNA and protein levels for the chloroplast proteins identified in our quantitative proteomics analysis, implying that the protein abundance changes are mediated posttranslationally. Overall, our ubiquitinomic and quantitative proteomic analyses indicated that CHLORAD functions much more broadly than was previously envisaged, by acting on many proteins of the chloroplast’s interior.

### Demonstrating CHLORAD involvement in the degradation of internal chloroplast proteins

**Fig. 4.**
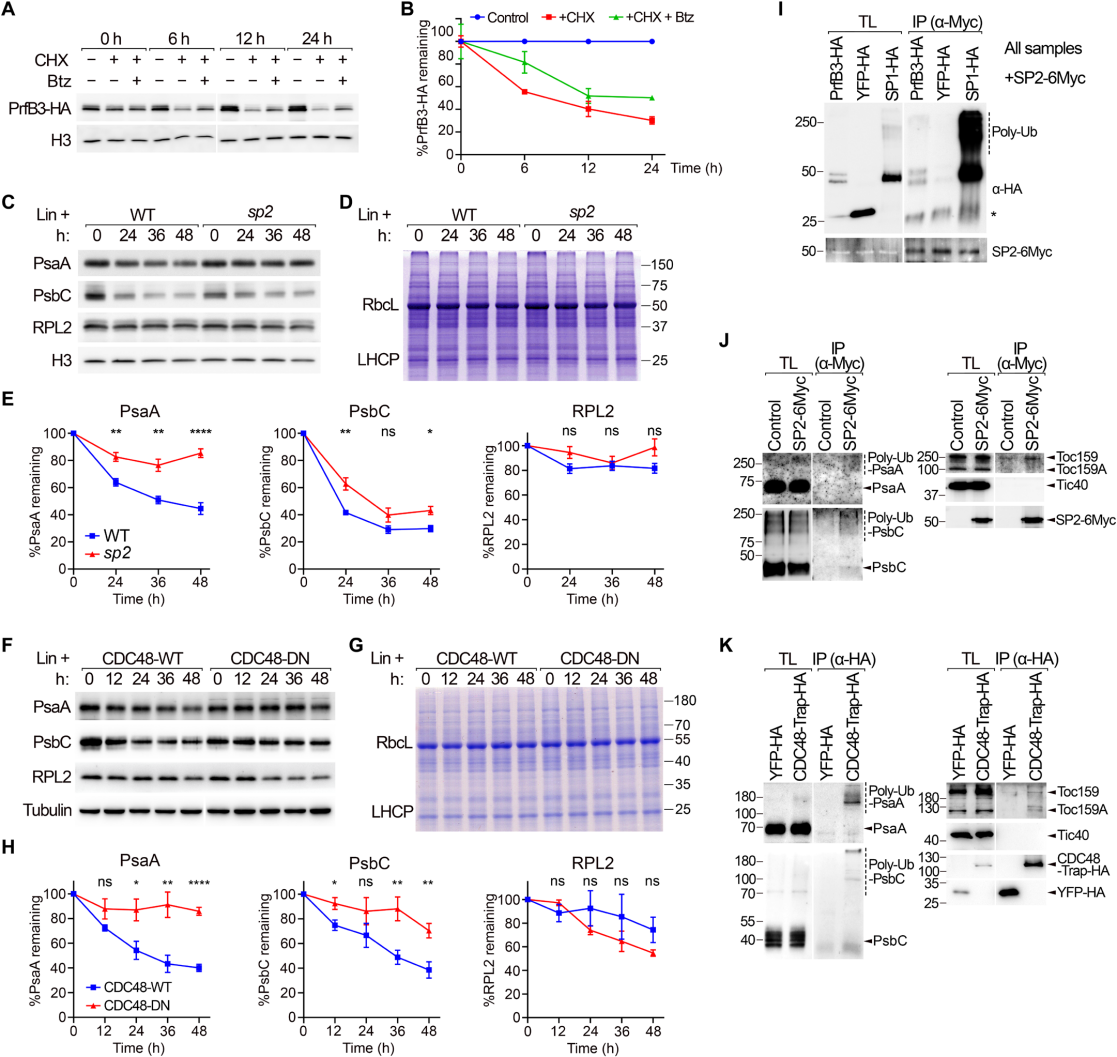
Proteins of the chloroplast interior are processed by CHLORAD. (**A** and **B**) Proteasome dependency of PrfB3 degradation was analyzed. Wild-type protoplasts expressing PrfB3-HA were incubated with/without cycloheximide (CHX) and proteasome inhibitor bortezomib (Btz) and analyzed by immunoblotting (A). Bands for PrfB3-HA were quantified and normalized to histone H3 control data (B). Time 0 was taken as 100%. Data are means ± SEM from at least two experiments. (**C** to **E**) SP2 dependency of chloroplast-encoded protein degradation was analyzed. Wild-type and *sp2* plants were incubated with lincomycin (Lin) and analyzed by immunoblotting (C) and Coomassie staining (D). Bands were quantified as in (B). Data are means ± SEM from at least four experiments. Asterisks in (E) indicate significance according to unpaired two-tailed Student’s *t* tests comparing the two genotypes at each time point (*P < 0.05, **P < 0.01, and ****P < 0.0001; ns, not significant). (**F** to **H**) CDC48 dependency of chloroplast-encoded protein degradation was analyzed. Data are equivalent to those in (C) to (E), except that CDC48-WT/DN transgenic plants were used. (**I** to **K**) Interaction of substrates with the CHLORAD apparatus was assessed by coimmunoprecipitation (co-IP). In (I), wild-type protoplasts were cotransfected with *SP2-6Myc* and either *PrfB3-HA*, *YFP-HA*, or *SP1-HA*. In (J), wild-type or SP2-6Myc seedlings were analyzed without protoplastation. In (K), wild-type protoplasts were transfected with *YFP-HA* or *CDC48-Trap-HA* [AtCDC48A^E581Q^ that stabilizes substrate binding (*8*)]. Immunoprecipitations used anti-Myc (I and J) or anti-HA (K) resin. YFP-HA acted as a negative control. Positions of molecular weight markers (sizes in kDa) are shown. Asterisk in (I) indicates a nonspecific band. TL, total lysate; poly-Ub, poly-ubiquitinated.

Identification of many internal chloroplast proteins (non-OEM proteins) as putative CHLORAD substrates was unexpected, as these proteins are separated from the cytosolic UPS apparatus by the envelope membranes. To confirm that these proteins are processed by the UPS, we examined their degradation following proteasome inhibition. We began with the nucleus-encoded stromal protein PrfB3. We monitored levels of a PrfB3–hemagglutinin (HA) fusion protein following inhibition of protein synthesis using cycloheximide and found that degradation was apparent within 6 hours, indicating brisk turnover. Crucially, degradation of PrfB3-HA was delayed following treatment with bortezomib that suppresses proteasome and CHLORAD activity (Fig. 4, A and B) (*8*).

Next, we analyzed the turnover of several chloroplast-encoded proteins in the CHLORAD-defective *sp2* and CDC48-DN backgrounds (*8*). In this case, native protein levels were monitored following treatment with lincomycin to inhibit chloroplast translation. The stabilities of PsaA and PsbC (which are both putative substrates and photosynthesis components) were clearly enhanced in the CHLORAD-defective genotypes (Fig. 4, C to H). In contrast, RPL2 (ribosomal protein L2) (a chloroplast-encoded ribosomal protein not identified as a putative CHLORAD substrate, used here as a control) was not stabilized in the *sp2* and CDC48-DN backgrounds. Thus, CHLORAD acts selectively on a diverse range of internal substrates, but not all chloroplast proteins.

To further establish a direct link between the CHLORAD apparatus and its putative targets in the chloroplast interior, we performed coimmunoprecipitation experiments. SP2-6Myc was found to specifically associate with PrfB3-HA, including high–molecular weight forms that we interpret to be polyubiquitinated (Fig. 4I). Similarly, SP2-6Myc and a CDC48 mutant with stabilized substrate binding (CDC48-Trap) (*8*) associated with two of the putative chloroplast-encoded CHLORAD substrates (PsaA and PsbC; Fig. 4, J and K, and fig. S9). Therefore, the action of CHLORAD on the stability of the internal substrates may be mediated through direct physical interactions.

### Demonstrating the role of CDC48 in the extraction of internal chloroplast proteins

We previously demonstrated that CDC48 drives the retrotranslocation of CHLORAD substrates from the OEM to the cytosol. To assess whether CDC48 is similarly involved in the extraction of substrates from the chloroplast interior, we performed in vivo retrotranslocation assays for two chloroplast-encoded substrates (PsaA and PsbC) using our established system (*8*). These substrates were selected to eliminate the possibility of confounding effects due to the accumulation of unimported preproteins in the cytosol.

**Fig. 5.**
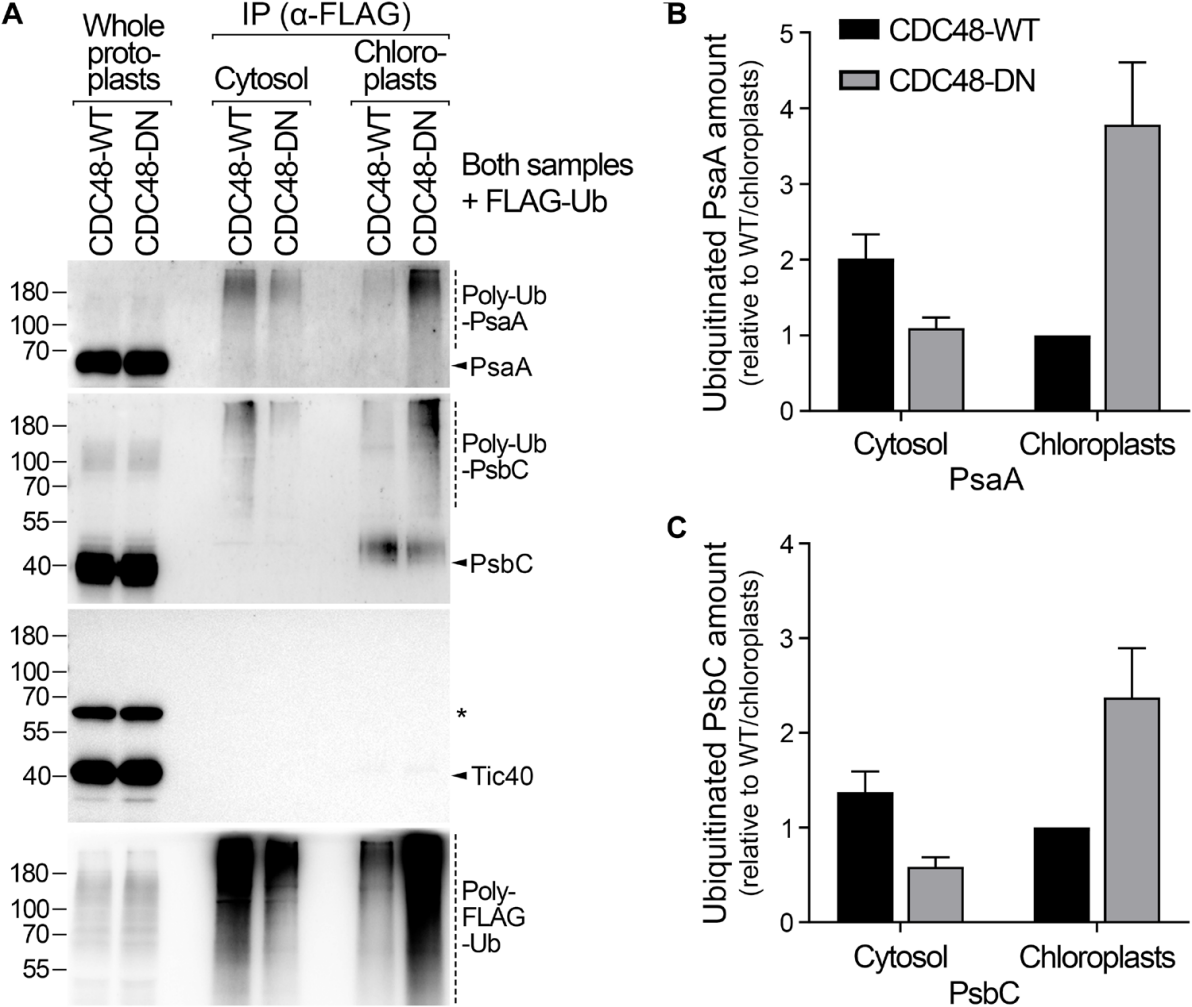
CDC48 is required for the extraction of polyubiquitinated photosynthesis proteins. (**A**) The role of CDC48 in the extraction of chloroplast-encoded PsaA and PsbC substrates was analyzed using an in vivo retrotranslocation assay. Protoplasts from CDC48-WT and CDC48-DN transgenic plants that were transiently expressing FLAG-Ub were treated, after estradiol induction, with bortezomib proteasome inhibitor and then separated into cytosol and chloroplast fractions. In this assay, retrotranslocation occurred in intact cells, and the extracted substrates were protected by bortezomib inhibition, which initiated the experiment. After fractionation, ubiquitinated proteins were immunoprecipitated from both fractions and detected by immunoblotting. Tic40, which is not a substrate of CHLORAD, served as a negative control. Typical immunoblotting results are shown. Positions of molecular weight markers (sizes in kDa) are shown to the left of the images. The asterisk indicates a nonspecific band. (**B** and **C**) Retrotranslocation efficiency was assessed by quantifying the relative amounts of ubiquitinated PsaA (B) and PsbC (C) in the cytosol and chloroplast fractions described in (A). Data are means ± SEM from at least three experiments.

Accordingly, we separated bortezomib-treated protoplasts from CDC48-DN and CDC48-WT plants into chloroplast and cytosol fractions and then enriched the ubiquitinated proteins from each fraction by immunoprecipitation. Resident and extracted PsaA and PsbC proteins, in the chloroplast and cytosol samples respectively, were then detected by immunoblotting (Fig. 5A). The extraction of polyubiquitinated PsaA and PsbC to the cytosol was clearly apparent, providing strong evidence that internal chloroplast protein can be exported from the organelle. Moreover, this extraction was inhibited in cells expressing CDC48-DN, as indicated by the deficiency of ubiquitinated material in the cytosolic fractions, pointing to an important role for CDC48 in the processing of such internal chloroplast proteins (Fig. 5).

### Evaluating the physiological importance of CHLORAD for photosynthesis and lipid homeostasis

**Fig. 6.**
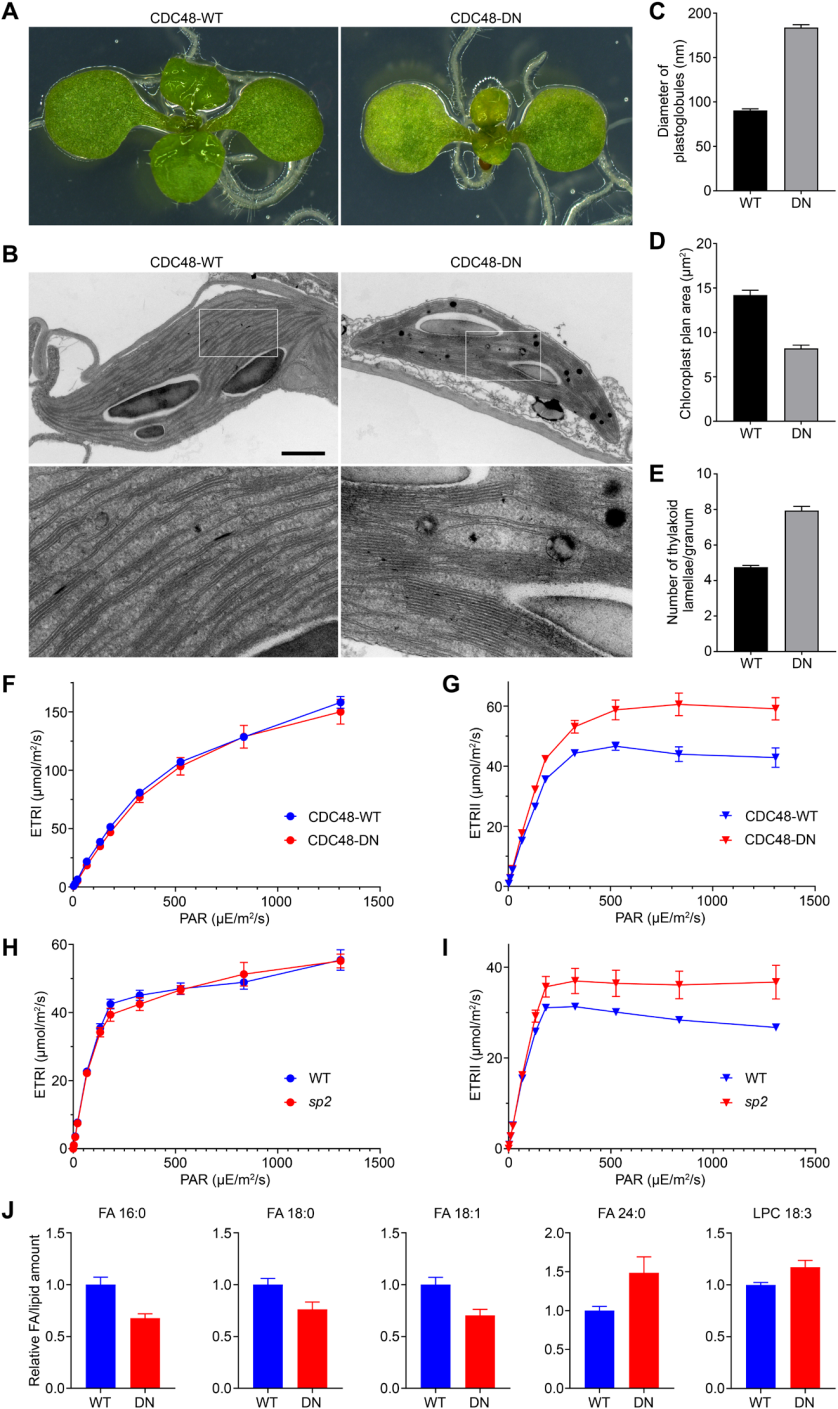
CHLORAD is required for normal photosynthetic function and lipid homeostasis. (**A** to **E**) Chloroplast development in 8-day-old CDC48-WT/DN seedlings, after 2-day estradiol induction, was studied. Cotyledons of typical plants (A) were analyzed by transmission electron microscopy (B). Upper images in (B) are at the same magnification (scale bar, 2 μm); higher-magnification (×4) images corresponding to the boxed regions are below. These micrographs were used to determine plastoglobule diameter (C), chloroplast cross-sectional area (D), and thylakoid lamellae per granum (E). Values are means ± SEM from 50 chloroplasts. (**F** to **I**) Photosynthetic electron flow through PSI [ETR(I)] and PSII [ETR(II)] in 4-week-old CDC48-WT and CDC48-DN plants (F and G), and in 5-week-old wild-type and *sp2* mutant plants (H and I), was measured. Here, the CDC48 plants received a 1-day estradiol induction. Plants were dark-adapted before exposure to actinic light [photosynthetically active radiation (PAR)] of the indicated intensities, followed by saturating pulses for the calculations. Values are means ± SEM from five to seven plants. (**J**) Free FA and polar lipid species in CDC48-WT/DN seedlings, after 2-day estradiol induction, were quantified. Data were normalized to internal standards and per unit fresh weight, and the normalized values were expressed relative to CDC48-WT (taken as 1). Numbers alongside the FA/lipid names describe the constituent FA chains, in the format (number of FA carbons):(number of FA double bonds). Selected FA/lipid species showing significant differences (*P* < 0.05) in CDC48-DN relative to CDC48-WT are shown; the complete dataset is in fig. S10. Values are means ± SEM from six biological replicates.

Short-term expression of CDC48-DN causes chlorosis (Fig. 6A) (*8*), which was previously linked to overaccumulation of reactive oxygen species due to a failure to properly regulate chloroplast protein import (*8*). This chlorosis also suggested morphological changes in the chloroplasts. To investigate the latter possibility, we assessed chloroplast ultrastructure in these plants. Chloroplasts in CDC48-DN plants contained enlarged plastoglobules (Fig. 6, B and C), which is consistent with oxidative stress (*30*) caused by disrupted photosystem component homeostasis, and were smaller than wild-type chloroplasts (Fig. 6, B and D), which can explain the chlorotic phenotype of the seedlings. Moreover, CDC48-DN chloroplasts contained larger grana [stacked thylakoids, where PSII is concentrated; (*31*)] and fewer stromal thylakoids (Fig. 6, B and E). These observations, alongside the data showing that CHLORAD acts selectively on a range of chloroplast-encoded photosystem components, suggested that CHLORAD plays a nuanced role in regulating the activities of the photosystems. To address this hypothesis, we simultaneously measured energy conversion in PSI and PSII by determining the electron transport rate (ETR) parameters ETR(I) and ETR(II), respectively (*32*), in mature CDC48-DN and *sp2* mutant plants, and corresponding control plants (Fig. 6, F to I). In this experiment, CDC48-DN (and CDC48-WT) expression was induced for an even shorter period to avoid large phenotypic changes that could have secondary consequences. Although the CDC48-DN and *sp2* plants did not display obvious visible differences from the respective control plants under these conditions, they both showed clearly elevated ETR(II) values, implying that CHLORAD normally acts to limit PSII activity.

Another critical role of chloroplasts is in lipid metabolism, as they are the major site of FA synthesis in plants (*33*). Two chloroplast envelope proteins involved in FA metabolism, FAX1 and LACS9, were identified as candidate CHLORAD substrates via ubiquitinomics (table S3) or quantitative proteomics (table S5). Both proteins are involved in FA export to the ER, and changes in their expression influence FA and lipid homeostasis (*34*, *35*), suggesting a role for CHLORAD in these processes. To investigate this possibility, we compared lipidomic profiles of CDC48-DN and CDC48-WT leaves, focusing on free FAs, glycerolipids, and glycerophospholipids. In total, 24 FA and lipid species were detected at significantly different levels in CDC48-DN (Student’s *t* test, *P* < 0.05). We observed decreases in chloroplast-produced FAs (16:0, 18:0, 18:1) and an increase in ER-elongated FA (24:0; Fig. 6J, fig. S10, and table S9), supporting the hypothesis that CHLORAD affects FA trafficking to the ER. Most lipid species were significantly reduced in CDC48-DN plants, with the exception of lysophosphatidylcholine (18:3) and phosphatidylglycerol (16:0/18:1; Fig. 6J, fig. S10, and table S9), which may reflect a general disruption of FA and lipid homeostasis linked to the loss of normal regulation by CHLORAD.

## DISCUSSION

Until recently, chloroplast proteolysis was thought to be dominated by internal chloroplast proteases of endosymbiotic origin (*3*, *4*). Then, our discovery of CHLORAD revealed an important role for the UPS in the degradation of OEM proteins (*7*, *8*). Internal chloroplast proteins were not considered to be likely targets of the cytosolic UPS, and of CHLORAD, due to the physical barrier presented by the double-membrane envelope. However, this work has now revealed how proteins of the chloroplast interior, including those in the IEM, stroma, and thylakoids, can be targeted by CHLORAD.

Proteomic evidence that chloroplast proteins are ubiquitinated has been presented previously (*26*, *36*, *37*). However, the relevant studies failed to determine whether the detected ubiquitination affects proteins that are resident in the organelle, which is highly pertinent given that most chloroplast proteins are synthesized in the cytosol before being imported into the organelle (*1*, *10*). The identified ubiquitinated proteins were most likely cytosolic preproteins, for three reasons. First, the proteins were extracted from whole seedlings or leaves, rather than from purified chloroplasts. Second, it is well known that unimported chloroplast preproteins in the cytosol are processed by the UPS via an Hsp70/CHIP (carboxyl terminus of Hsc70-interacting protein)–dependent pathway (*25*, *38*). Third, no chloroplast-encoded proteins were identified as being ubiquitinated in those studies, which is especially noteworthy given that such proteins are extremely abundant.

Here, we conclusively demonstrated that chloroplast-resident proteins, including those of internal chloroplast compartments, are ubiquitinated and degraded by the proteasome. Evidence for internal ubiquitination included thermolysin treatment of purified chloroplasts and chloroplast subfractionation. Furthermore, ubiquitinomic analysis of isolated chloroplasts identified 316 chloroplast proteins with clear ubiquitination sites, many from internal compartments, and of these, 13 were chloroplast-encoded proteins, ruling out the possibility that they were modified in the cytosol as precursors. This was consistent with a recent report showing that two of these proteins, RbcL and AtpB, are regulated by a CDC48 complex, although ubiquitination sites were not identified (*39*). Our results were supported by quantitative proteomics, steady-state protein abundance and turnover data, and physiological analyses, which all together robustly demonstrated that ubiquitination affects the chloroplast’s proteome and functions extensively. Apart from the well-established targets in the TOC apparatus (*7*, *8*), the substrates of CHLORAD identified here are involved in diverse processes including photosynthesis, FA metabolism, and chloroplast gene expression.

An obvious question arising from these results concerns how internal chloroplast proteins are delivered to the cytosol for degradation. A retrotranslocation system comprising SP2 and CDC48 was previously shown to mediate the extraction of OEM substrates (*8*), and we present evidence here that the same components act on internal substrates as well. First, the steady-state levels and turnover rates of internal chloroplast proteins were affected by SP2 and CDC48. Second, internal chloroplast proteins interacted physically with SP2 and CDC48. Third, in vivo retrotranslocation assays showed that CDC48 is required (presumably as a molecular motor) for the extraction of ubiquitinated internal proteins to the cytosol. However, exactly how stromal and thylakoidal proteins traverse the IEM remains unknown, as both SP2 and CDC48 operate at the OEM. This issue highlights a fundamental difference between ERAD (*14*, *40*), which acts at a single membrane, and CHLORAD, which acts on endosymbiotic organelles with two bounding membranes. Although inner membrane retrotranslocation systems have not been identified, in either chloroplasts or mitochondria, our results indicate that such a system may exist in chloroplasts, and that there may even be internal ubiquitination mechanisms. With regard to the latter point, ubiquitination likely involves previously unidentified mechanisms as the established CHLORAD E3 ligase, SP1, is apparently not involved (fig. S11).

Photosynthesis, and its regulation, has been studied intensively for decades owing to its fundamental importance and iconic status as a defining feature of plants. Proteolysis is one of the key mechanisms for maintaining photosynthetic performance (*3*, *4*). Turnover of internal chloroplast proteins involves multiple protease types, including three of prokaryotic origin: FtsH, Deg, and Clp. For example, PSII core subunit D1 is frequently degraded by FtsH and Deg, as its reaction center role renders it highly susceptible to photooxidative damage, necessitating frequent replacement. Other PSII core subunits, such as PsbB, PsbC, and D2 (PsbD), are more stable and might only be degraded under stress conditions, although the mechanisms involved are less clear (*41*). Even less information exists concerning the degradation of PSI subunits. The present study sheds light on PSI and PSII degradation, as it reveals an important role in photosynthetic regulation for the most pervasive proteolytic system in eukaryotes, the UPS, which is mediated by targeting key photosystem proteins including PsaA and PsbC.

Among the 24 photosystem (PSI and PSII) components identified in our ubiquitinome analysis, six are synthesized by the chloroplast itself. Analyses of the ubiquitination and turnover of PsaA and PsbC identified these proteins as bona fide CHLORAD substrates, while the importance of this regulation was supported by data on photosynthetic performance and chloroplast ultrastructure in CHLORAD mutants. CHLORAD influenced the balance of activity between PSII and PSI, suggesting that the regulatory pathway allows for nuanced control. The fact that ETR(II) is specifically elevated in the CHLORAD mutants also implies that levels of PSII components (such as PsbC) are normally limiting for PSII activity. Why photosystem and other chloroplast components are degraded by both organellar proteases and the UPS is unclear. One possibility is that the proteases function mainly in damage-induced responses, whereas the UPS acts to regulate specific substrates. Alternatively, the different systems might act at different stages or in response to different cues. For instance, D1 requires frequent turnover in the light whenever photosynthesis is active (*5*, *6*, *42*), so use of thylakoid-localized proteases may be more economical and responsive to nearby chloroplast signals. In contrast, CHLORAD may be more suitable for the regulation of stable substrates that require infrequent degradation under conditions communicated by extrachloroplastic signals. Last, centralized control by the nucleocytosolic system may enable better integration of chloroplast functions within the cellular repertoire so that activities across different compartments can be more closely coordinated.

Circumstances warranting CHLORAD action may include abiotic stress (*13*) or those developmental phases during which internal organelle membranes are remodeled, such as de-etiolation (when the light-triggered degradation of prolamellar bodies occurs in etioplasts) or fruit ripening (when the disassembly of thylakoids occurs in developing chromoplasts) (*7*, *15*). It is evident that protein degradation is crucial in these situations, but the underlying mechanisms are unclear. Our previous work revealed that CHLORAD is involved by regulating the protein import machinery (*11*, *12*); however, the new results reported here indicate that CHLORAD can also act more directly and that its established role in import regulation was arguably just the tip of the iceberg. For example, the maintenance of photosynthetic performance under senescence-inducing conditions in *sp2* mutants could be a consequence of reduced degradation of the photosystems, in addition to altered protein import (*8*). Aside from the photosystem components, a number of other chloroplast-encoded photosynthesis proteins (i.e., one cytochrome b_6_f subunit, three ATP synthase subunits, one NDH complex subunit, and the RuBisCO large subunit) were found to be ubiquitinated. Thus, it appears that CHLORAD can regulate diverse aspects of photosynthesis, including electron transport, energy transduction, and carbon fixation.

Ubiquitination sites were identified in 21 chloroplast-localized proteins with FA/lipid-related functions, while the abundance of six such proteins was affected by CDC48. These data point to an important and direct role for CHLORAD in the regulation of plant FA/lipid metabolism. One of the relevant proteins is FAX1, an IEM protein involved in FA export from chloroplasts that is required for normal cellular FA and lipid homeostasis. In *fax1* mutants, chloroplast-synthesized FAs (C_16–18_) are increased, whereas very-long-chain FAs (which are elongated in the ER and thus require the export of C_16–18_ FAs) are reduced, with various developmental consequences (*35*). Our results identify FAX1 as a target of CHLORAD and link the impairment of CHLORAD-dependent degradation to perturbations in cellular FA/lipid homeostasis. Moreover, the pattern of FA disturbances observed in CDC48-DN plants was very similar to that seen previously in FAX1-overexpressing plants (*35*). It is noteworthy that carbohydrate metabolism and lipid metabolism are closely connected and that the flow of carbon into oil (via FAX1) must be strictly controlled (*43*). Our results point to an important role for CHLORAD at this crucial metabolic nexus, the regulation of which is not currently well understood.

In this work, we systematically identified the targets and ubiquitination sites of CHLORAD. Our results reveal that CHLORAD acts directly on a diversity of chloroplast proteins, extending far beyond the TOC apparatus to the organelle’s interior, and that the UPS broadly influences chloroplast functions including photosynthesis and lipid metabolism. Furthermore, the data illustrate how chloroplast proteostasis is remarkably complex and chimeric in nature, incorporating ancient systems inherited from the ancestral endosymbiont and eukaryotic ubiquitin-dependent processes. These findings also provide opportunities for cultivated plant improvement (e.g., by maximizing photosynthetic activity) (*44*) and thus may contribute to global challenges such as food security and carbon neutrality.

## MATERIALS AND METHODS

### Plant growth conditions

All *Arabidopsis thaliana* plants were of the Columbia-0 (Col-0) ecotype. The *sp2-4* mutant, transgenic lines expressing dominant-negative mutant and wild-type control versions of CDC48 with a C-terminal FLAG tag driven by an estradiol-inducible promoter (CDC48-DN and CDC48-WT, respectively), and SP2 with a C-terminal 6× Myc tag driven by the 35*S* promoter (SP2-6Myc) have all been described previously (*8*). All plants were grown under a long-day cycle (16-hour light/8-hour dark), essentially as described previously (*45*). For in vitro growth, seeds were surface-sterilized, sown on Murashige-Skoog (MS) agar medium in petri plates, cold-treated at 4°C, and thereafter kept in a growth chamber, as described previously (*45*). For the induction of CDC48-DN or CDC48-WT expression in the corresponding transgenic lines, 8-day-old plants were transferred onto MS medium supplemented with 4 μM estradiol (Sigma-Aldrich) and incubated for an additional 2 days.

### Measurement of photosynthetic electron flow

Photosynthetic parameters ETR(I) and ETR(II), which indicate electron flow through PSI and PSII, were measured on developmentally equivalent mature leaves (the third pair of true leaves) of 4- or 5-week-old plants using a DUAL-PAM-100 (Walz) in the Fluorescence and P700 Measure Mode of a dual channel. For the measurements, plants were dark-adapted for 20 min at 22°C before the actinic light intensity was increased stepwise from 0 to 1300 μmol photons m^−2^ s^−1^. After exposure to each light intensity for 1 min, a 0.8-s pulse of saturating light (8000 μmol photons m^−2^ s^−1^) was applied, and the maximum PSII and PSI quantum yields, *F*_m_′ and *P*_m_′, respectively, were simultaneously recorded. Then, the stable PSII and PSI quantum yields, *F*_s_ and *P*_s_, were concurrently recorded. ETR(I) and ETR(II) were calculated as follows: 0.42 × PFD × (*P*_m_′ − *P*_s_)/*P*_m_ and 0.42 × PFD × (*F*_m_′ − *F*_s_)/*F*_m_′, respectively, where PFD is the photon flux density of actinic light, as described previously (*46*). At least five leaves (each one from a different plant) were analyzed per genotype in each experiment.

### Plasmid constructs

All primers used are listed in table S10. The *SP1-HA*, *YFP-HA*, *FLAG-Ub*, *CDC48-Trap-HA*, *Tic110-YFP*, and *Toc33-HA* constructs have all been described previously (*7*, *8*). All *Arabidopsis* coding sequences (CDSs) were polymerase chain reaction–amplified from Col-0 cDNA. The Gateway cloning system (Invitrogen) was used to make most of the constructs, and all entry clones were verified by DNA sequencing. Constructs for the estradiol-inducible expression of untagged CDC48-DN or CDC48-WT (*CDC48-DNa* and *CDC48-WTa*, respectively) were generated using the pMDC7 binary vector (*8*), essentially as described previously (*8*) except without addition of the FLAG tag. To generate N-terminally 6× Myc-tagged ubiquitin (6Myc-Ub), the ubiquitin CDS was amplified from the *AtUBQ11* gene (At4g05050), cloned into the pE3n vector (*47*), and then subcloned into the pB2GW7 35*S*-driven expression vector (*48*) for stable plant transformation (generating the *6Myc-Ub* construct). The CDSs of LACS9 (At1g77590), FAX1 (At3g57280), and CP12 (At3g62410) were cloned into the p2GWY7 plant expression vector (*48*) providing a C-terminal YFP tag (generating the *LACS9-YFP*, *FAX1-YFP*, and *CP12-YFP* constructs). To generate HA-tagged PrfB3 (At3g57190) and SP2 (At3g44160), the corresponding CDSs were cloned into a modified p2GW7 vector (*8*) providing a C-terminal HA tag (generating the *PrfB3-HA* and *SP2-HA* constructs).

### Transient assays and stable plant transformation

Protoplast isolation and transient assays were carried out as described previously (*8*). When required, bortezomib [Selleckchem; prepared as a 10 mM stock solution in dimethyl sulfoxide (DMSO)] was added to the protoplast culture medium at 15 hours following transfection to a final concentration of 5 μM; subsequently, the culture was incubated for a further 2 to 3 hours before analysis. When using protoplasts isolated from the CDC48-WT and CDC48-DN transgenic lines, 10 μM estradiol (prepared as a 10 mM stock solution in ethanol) was included in the culture medium throughout the incubation of protoplasts. For YFP fluorescence and immunoprecipitation assays, 0.1 (10^5^ cells) or 1 ml (10^6^ cells) aliquots of protoplasts were transfected with 5 or 100 μg of DNA, respectively; the fluorescence signals were analyzed after 15 to 18 hours.

Transgenic lines carrying the *CDC48-DNa*, *CDC48-WTa*, and *6Myc-Ub* constructs were generated by *Agrobacterium*-mediated transformation (*49*, *50*). Transformants were selected using MS medium containing hygromycin B (50 μg/ml; Melford) or phosphinothricin (10 μg/ml; Duchefa). Approximately 10 independent T_2_ lines were analyzed per construct, and one representative line with a single T-DNA insertion (which showed a 3:1 segregation on selective MS medium in the T_2_ generation) was chosen for further analysis.

### Microscopy

Transmission electron microscopy was performed as described previously (*49*). For induction of CDC48-WT or CDC48-DN expression in the corresponding transgenic lines, 8-day-old plants were transferred onto MS agar medium supplemented with 4 μM estradiol and incubated for an additional 2 days. Measurements were recorded using at least 50 different plastids per genotype and were representative of three individuals per genotype. Chloroplast cross-sectional area was estimated as described previously (*49*, *51*) using the equation: π × 0.25 × length × width. Numbers of thylakoid lamellae per granal stack were counted as previously described (*7*, *49*) for at least 180 resolvable grana from three individuals per genotype. Plastoglobule diameter was determined by measuring at least 150 different plastoglobules from three individuals per genotype.

All fluorescence microscopy experiments were conducted at least twice with the same results, and typical images are presented. The imaging of YFP and chlorophyll fluorescence signals was conducted by examining protoplasts using a Zeiss LSM 510 META laser scanning confocal microscope (Carl Zeiss Ltd.), as described previously (*13*). All images were captured using the same settings to enable comparisons.

### Chloroplast isolation, protease treatment, and subfractionation

Chloroplasts were isolated from plants grown in vitro for 8 to 10 days (or, when stated, from protoplasts). For CDC48-DN and CDC48-WT, 8-day-old plants were transferred onto MS agar medium (without sucrose) supplemented with 4 μM estradiol and incubated for an additional 2 days before chloroplast isolation. For 6Myc-Ub, 8-day-old plants were transferred to MS liquid medium (without sucrose) supplemented with 5 μM bortezomib and incubated for an additional 2 days before chloroplast isolation. Chloroplast isolations and protease treatments were performed as described previously (*8*, *45*).

For chloroplast subfractionation, pelleted chloroplasts from 6Myc-Ub plants were first resuspended and lysed in prechilled hypotonic lysis buffer [25 mM Hepes-KOH (pH 8.0), supplemented with 0.5% plant protease inhibitor cocktail (PPIC; Sigma-Aldrich, P9599)] in a rotator for 30 min at 4°C. Then, the lysate was centrifuged at 18,000*g* for 30 min at 4°C. The recovered supernatant was centrifuged again at 18,000*g*, for 30 min at 4°C, and the resulting supernatant (soluble fraction) was retained for further analysis. The pellet of the first centrifugation step was resuspended in prechilled hypotonic lysis buffer and centrifuged at 18,000*g* for 30 min at 4°C. The pellet resulting from this centrifugation step (membrane fraction) was retained for further analysis.

### SDS-PAGE, immunoblotting, and immunoprecipitation

SDS–polyacrylamide gel electrophoresis (SDS-PAGE) and immunoblotting were performed essentially as described before (*51*, *52*). Primary antibodies were as follows. To detect TOC proteins or components of the translocon at the IEM of chloroplasts, we used the following: anti–atToc75-III antibody (*50*), anti-atToc159 antibody (*53*), anti-atToc33 (G-domain) antibody (*50*), anti-atTic110 antibody (*54*), and anti-atTic40 antibody (*50*). To detect non-TOC OEM proteins, we used the following: anti-CHUP1 antibody (*55*) and anti-FtsZ2 antibody (*56*). To detect chloroplast stromal proteins, we used the following: anti-RPL2 [HUABIO, PAB20010; or as described previously (*57*)], anti-COR15 antibody (*58*), anti-RbcL antibody (*59*), and anti-PAO antibody (*60*). To detect chloroplast thylakoid proteins, we used the following: anti-PsaA antibody [HUABIO, PAB01001; or as described previously (*61*)], anti-PsbC (CP43) antibody (HUABIO, PAB02003), anti-PsbD antibody (Agrisera, AS06146), and anti-LHCP antibody (*49*, *50*). To detect proteins of other cellular compartments, we used the following: anti–α-tubulin (cytosol; HUABIO, T5168) and anti-H3 histone (nucleus; Abcam, ab1791). Other primary antibodies that we used were as follows: anti-HA tag antibody (Sigma-Aldrich, H6908), anti–c-Myc tag antibody (Abcam, ab9106), anti–green fluorescent protein (GFP) antibody (detects both GFP and YFP; Sigma-Aldrich, SAB4301138), and anti-FLAG tag antibody (Sigma-Aldrich, F7425). We used Tic110, α-tubulin, or H3 as loading controls.

Secondary antibodies were polyclonal goat anti-rabbit immunoglobulin G (IgG) conjugated with horseradish peroxidase (Sigma-Aldrich, 12-348) or, in the case of immunoprecipitations, monoclonal mouse anti-rabbit IgG light chain–specific conjugated with peroxidase (Jackson ImmunoResearch, 211-032-171). Chemiluminescence was detected using EZ-ECL (Biological Industries, Sartorius) or ECL Plus Western Blotting Detection Reagents (GE Healthcare) and an LAS-4000 imager (Fujifilm). Band intensities were quantified using ImageJ (*62*) or Aida software (Raytest). Quantification data were based on results from at least three experiments all showing a similar trend. Typical images are shown in all figures.

For the immunoprecipitation of HA-tagged proteins, total protein (~500 mg) was extracted from protoplasts in immunoprecipitation (IP) buffer [25 mM tris-HCl (pH 7.5), 150 mM NaCl, 1 mM EDTA, and 1% Triton X-100] containing 0.5% PPIC (Sigma-Aldrich) and centrifuged at 20,000*g* for 10 min at 4°C. The clear lysate was then incubated with 50 μl of EZview Red Anti-HA Affinity Gel (Sigma-Aldrich) for 2 hours to overnight at 4°C with slow rotation. After six washes with 500 μl of IP-washing buffer [25 mM tris-HCl (pH 7.5), 150 mM NaCl, 1 mM EDTA, and 0.5% Triton X-100], bound proteins were eluted by boiling in 2× SDS-PAGE loading buffer [50 mM tris-HCl (pH 6.8), 20% glycerol, 1% SDS, and 0.1 M dithiothreitol (DTT)] for 5 min and analyzed by SDS-PAGE and immunoblotting. A similar procedure was adopted for the immunoprecipitation of Myc- or FLAG-tagged proteins, except that 50 μl of EZview Red Anti-c-Myc Affinity Gel (Sigma-Aldrich) or Anti-FLAG M2 Affinity Gel (Sigma-Aldrich) was used instead of the anti-HA gel. When detecting ubiquitinated proteins, the IP buffer also contained 10 mM *N*-ethylmaleimide (NEM; Sigma-Aldrich).

To assess in vivo ubiquitination of chloroplast substrates, FLAG-Ub was transiently overexpressed in protoplasts to increase detection sensitivity for higher–molecular weight forms (*8*). Protoplasts were lysed in denaturing buffer [25 mM tris-HCl (pH 7.5), 150 mM NaCl, 5 mM EDTA, 10 mM NEM, 1% SDS, 2% Sarcosyl, and 5 mM DTT], before incubation at 75°C and 600 rpm for 30 min in a Thermomixer Comfort (Eppendorf). The lysate was then diluted by adding 1 volume of 2% Triton X-100 and 8 volumes of IP buffer containing 0.5% PPIC and incubated on ice for 30 min. Ubiquitinated proteins were enriched through subsequent immunoprecipitation steps as described above with Anti-FLAG M2 Affinity Gel. Immunoblot analysis of the precipitates using antibodies against proteins of interest was used to demonstrate protein ubiquitination, as indicated by higher molecular weight smears.

### Mass spectrometry analysis of Myc-tagged ubiquitin interactors

Eight-day-old 6Myc-Ub plants were transferred to liquid MS medium (without sucrose) supplemented with 5 μM bortezomib and incubated for an additional 2 days before chloroplast isolation. Isolated chloroplasts were subjected to immunoprecipitation using EZview Red Anti-c-Myc Affinity Gel (see above). Elution of immunoprecipitated proteins was performed using c-Myc peptide (100 μg/ml; Sigma-Aldrich, M2435), on ice for 15 min, and eluates were collected following centrifugation at 8200*g* for 30 s at 4°C.

Protein digestion was performed using a filter-aided sample preparation protocol. Eluate (50 μl) was denatured, alkylated with 950 μl of urea buffer [8 M urea, 100 mM ammonium bicarbonate (AB), 10 mM tris(2-carboxyethyl)phosphine hydrochloride, 50 mM 2-chloroacetamide, and 0.2% protease inhibitor (Sigma-Aldrich, P8340)], and incubated in the dark at room temperature for 30 min. Filters (30 kDa cutoff; Vivacon 500) were prewashed [using 0.1% (v/v) trifluoroacetic acid (TFA) and 50% (v/v) acetonitrile] and then loaded with 200 μl of 8 M urea in 100 mM AB, followed by 100 μl of the denatured sample and an additional 100 μl of 8 M urea in 100 mM AB. Filters were centrifuged at 14,000*g* for 15 min and then washed twice using 6 M urea in 25 mM AB. LysC protease (Promega, V1671; 200 ng in 50 μl of 6 M urea and 25 mM AB) was added to each filter before incubation at 37°C for 4 hours. The digestion solution was diluted to <1 M urea upon addition of trypsin (Promega, V511A; 200 ng in 300 μl of 1 M urea, 25 mM AB, and 1 mM CaCl_2_) before incubation at 37°C overnight. After digestion, the eluate fractions were collected by centrifugation at 14,000*g* for 15 min at 25°C. The filters were washed using 0.1% TFA, then 0.1% TFA, and 50% acetonitrile, and the flow-through fractions were combined with the initial eluate fractions and dried down in a SpeedVac concentrator.

Resulting tryptic peptides were analyzed on an EASY-nLC 1000 system (Thermo Fisher Scientific) connected to a Q Exactive mass spectrometer (Thermo Fisher Scientific) through an EASY-Spray nanoelectrospray ion source (Thermo Fisher Scientific). The peptides were initially trapped on a C18 PepMap100 precolumn [300 μm inner diameter (i.d.) by 5 mm, 100-Å pore size; Thermo Fisher Scientific] using solvent A (0.1% formic acid in water). Trapped peptides were separated on an in-house constructed analytical column (75 μm i.d. by 500 mm, Reprosil C18, 1.9-μm particle size, 100-Å pore size) using a linear gradient [length: 60 min, 18 to 30% solvent B (0.1% formic acid and 5% DMSO in acetonitrile); flow rate: 200 nl/min]. The separated peptides were electrosprayed directly into the mass spectrometer operating in a data-dependent mode. Full-scan MS spectra were acquired in the Orbitrap [scan range of 350 to 1500 mass-to-charge ratio (*m/z*), resolution of 70,000, automatic gain control (AGC) target of 3 × 10^6^, and maximum injection time of 50 ms]. After the MS scans, the 10 most intense peaks were selected for high-energy collisional dissociation (HCD) fragmentation at 30% of normalized collision energy. HCD spectra were also acquired in the Orbitrap (resolution of 17,500, AGC target of 5 × 10^4^, and maximum injection time of 120 ms).

Protein identification and quantification were performed using the Andromeda search engine implemented in MaxQuant (1.6.3.4). Peptides were searched against a reference proteome of *A. thaliana* (UniProt database, downloaded Jan 2017) with the custom addition of the 6× Myc-tagged ubiquitin sequence. The di-Gly modification at lysine was added as an additional variable modification, while other settings were kept at default parameters (*63*). False discovery rate (FDR) was set at 1% for both peptide and protein identification.

### Di-Gly ubiquitinome analyses

Ubiquitinome analyses were performed using isolated chloroplast samples from wild-type and CDC48-DN plants. Wild-type samples were analyzed in the Advanced Proteomics Facility, University of Oxford, and CDC48-DN samples were analyzed by Shanghai Applied Protein Technology Co. Ltd.

Isolated wild-type chloroplasts were lysed in urea buffer [8 M urea, 100 mM AB, 10 mM tris(2-carboxyethyl)phosphine hydrochloride, and 50 mM 2-chloroacetamide] containing 1% PPIC at room temperature for 30 min in the dark. The lysate was cleared by centrifugation at 4500*g* for 15 min at room temperature, and then, the concentration of urea in the supernatant was diluted to 6 M using 25 mM AB. LysC protease (Wako, 129-02541) was added [to a LysC:protein ratio of 1:100 (w/w)], and digestion was performed at 37°C for 4 hours with mixing. The urea concentration was diluted to 1 M using 25 mM AB, and then, CaCl_2_ was added to a final concentration of 1 mM. Next, trypsin (Sigma-Aldrich, T-1426) was added [to a trypsin:protein ratio of 1:40 (w/w)], and digestion was performed at 37°C overnight with mixing. Trifluoroacetic acid was added (to a final concentration of 1%); precipitation was allowed to occur for 15 min on ice, and the precipitates were removed by centrifugation at 1780*g* for 15 min at room temperature. Peptides in the supernatant were then purified using Sep-Pak C_18_ columns (Waters, WAT051910). For analysis of di-Gly modification–enriched peptides, an immunoprecipitation step was performed using the PTMScan Ubiquitin Remnant Motif (K-ε-GG) Kit (Cell Signaling Technology) according to the manufacturer’s instructions.

Liquid chromatography–tandem mass spectrometry (LC-MS/MS) analysis was conducted as described above (for the analysis of Myc-tagged ubiquitin interactors), with the following changes: The peptides were analyzed on a nanoUHPLC (Thermo Fisher Scientific). Trapped peptides were separated on an EASY-Spray Acclaim PepMap analytical column (75 μm i.d. by 500 mm, RSLC C18, 2-μm particle size, 100-Å pore size; Thermo Scientific) using a linear gradient [length: 60 min; 15 to 35% solvent B (0.1% formic acid and 5% DMSO in acetonitrile); flow rate: 200 nl/min].

Protein identification and quantification were performed using Sequest HT in Proteome Discoverer 1.4 (Thermo Fisher Scientific, version 1.4.0.288). Tandem mass spectra were searched against a database containing 16,480 protein entries from *A. thaliana* (UniProt, release from July 2022) and common contaminants. During database searches, it was considered that cysteines were fully carbamidomethylated (+57.0215, statically added), methionines were fully oxidized (+15.9949, dynamically added), all N-terminal residues were acetylated (+42.0106, dynamically added), and lysines were ubiquitinated (di-Gly, +114.043, dynamically added). Two missed cleavages were permitted. FDR was set at 1% for both peptide and protein identification. Protein identification and quantification were also conducted as described above (for the analysis of Myc-tagged ubiquitin interactors), without the custom addition of the 6× Myc-tagged ubiquitin sequence to the database.

For analysis of di-Gly modification–enriched peptides of CDC48-DN samples, isolated chloroplasts were lysed in urea buffer [8 M urea and 100 mM tris-HCl (pH 8.5)] for protein extraction. Then, DTT was added to a final concentration of 10 mM, and the samples were mixed at 600 rpm for 1.5 hours at 37°C and cooled to room temperature before being subjected to trypsin digestion and desalting. Desalted peptides (~2 mg) were reconstituted in 1.4 ml of precooled immunoaffinity purification (IAP) buffer [50 mM MOPS (pH 7.2), 10 mM sodium phosphate, and 50 mM NaCl] and then incubated with anti–K-ε-GG antibody beads [PTMScan Ubiquitin Remnant Motif (K-ε-GG) Kit, Cell Signaling Technology] at 4°C for 1.5 hours. The antibody beads were collected by centrifugation at 2000*g* for 30 s and then washed three times with 1 ml of precooled IAP buffer and three times with precooled water. The di-Gly peptides were eluted with 40 μl of 0.15% TFA after incubation at room temperature for 10 min; the elution was repeated once, and the eluates were combined. After centrifugation at 2000*g* for 30 s, supernatants were collected and desalted using Millipore C18 StageTips (Sigma-Aldrich, ZTC18S096).

LC-MS/MS analysis of CDC48-DN samples was performed using a Q Exactive mass spectrometer (Thermo Fisher Scientific) that was coupled to an EASY-nLC (Thermo Fisher Scientific) for 60, 120, or 240 min. The peptides were loaded onto a reversed-phase trap column (Acclaim PepMap100, 100 μm i.d. by 2 cm, nanoViper C18; Thermo Scientific) connected to the C18 reversed-phase analytical column (EASY-Column, 75 μm i.d. by 10 cm, 3-μm particle size; Thermo Scientific) in buffer A (0.1% formic acid) and separated with a linear gradient of buffer B (84% acetonitrile and 0.1% formic acid) at a flow rate of 300 nl/min controlled by IntelliFlow technology. The mass spectrometer was operated in positive ion mode. MS data were acquired using a data-dependent top 10 method, dynamically choosing the most abundant precursor ions from the survey scan (300 to 1800 *m/z*) for HCD fragmentation. AGC target was set to 3 × 10^6^, and maximum inject time was set to 10 ms. Dynamic exclusion duration was 40.0 s. Survey scans were acquired at a resolution of 70,000 at 200 *m/z*; resolution for HCD spectra was set to 17,500 at 200 *m/z*, and isolation width was 2 *m/z*. Normalized collision energy was 30 eV, and the underfill ratio, which specifies the minimum percentage of the target value likely to be reached at maximum fill time, was defined as 0.1%. The instrument was run with peptide recognition mode enabled.

The MS raw data for each sample were combined and searched using the MaxQuant software (*64*) against an *Arabidopsis* UniProt database (*65*) for chloroplast (plastid)–associated proteins. Trypsin was chosen as the enzyme with a maximum of two missed cleavages allowed. Precursor and fragment mass error tolerances were set at 20 parts per million (ppm) and 0.1 Da, respectively. Peptide modifications allowed during the search were as follows: carbamidomethyl (C, fixed), GlyGly (K, variable), and oxidation (M, variable). All matched MS/MS spectra were filtered by mass accuracy and matching scores to reduce protein FDR to ≤1% on the basis of the target-decoy strategy using a reversed database.

### Quantitative proteomic analysis

Eight-day-old CDC48-DN and CDC48-WT plants were transferred onto MS agar medium supplemented with 4 μM estradiol and incubated for a further 2 days before whole seedlings were collected, weighed, and frozen in liquid N_2_. Three independent samples of each genotype were collected. Quantitative proteomic analysis of these whole-seedling samples was performed by Shanghai Luming Biotechnology Co. Ltd. Frozen seedling samples (500 mg each) were ground to a power and then further ground in 1 ml of extraction buffer [250 mM tris-HCl, 0.7 M sucrose, 100 mM NaCl, 50 mM EDTA, and 10 mM DTT (pH 7.8); freshly prepared]. The samples were then mixed with an equal volume of tris-buffered phenol for 30 min at 4°C, before centrifugation at 7100*g* for 10 min at 4°C to collect the upper phenolic phase. The supernatants were mixed with five volumes of 0.1 M cold ammonium acetate/methanol solution and precipitated at −20°C overnight. Then, the proteins were pelleted by centrifugation at 12,000*g* at 4°C for 10 min (all centrifugation steps below were similar). Pellets were washed twice with ice-cold methanol and then twice more with ice-cold acetone; after each washing step, the samples were pelleted by centrifugation. The final pellets were dried at room temperature for 3 min and resuspended in SDS lysis buffer (Beyotime, Shanghai) for 3 hours. Last, the samples were centrifuged two further times, and the supernatants were retained.

The filter-aided sample preparation method (*66*) was used for proteolysis. Briefly, 100 μg of each sample was placed in an Amicon ultrafiltration centrifuge tube (Merck, 10 kDa molecular weight cutoff), 120 μl of reductant buffer [10 mM DTT, 8 M urea, and 100 mM triethylammonium bicarbonate (TEAB) (pH 8.0)] was added, and the mixture was incubated for 1 hour at 60°C. Iodoacetamide was added to a final concentration of 50 mM, and the solution was incubated in darkness at room temperature for 40 min. After centrifugation into a collection tube, the flow-through solution was discarded. Then, 100 μl of 300 mM TEAB buffer was added to the ultrafiltration tube, followed by 3 μl of sequencing-grade trypsin solution (1 μg/μl; Promega), and the sample was incubated for 12 hours at 37°C. Digested peptides were collected as the flow-through in a new collection tube by centrifugation. Then, 50 μl of 200 mM TEAB solution was added to the ultrafiltration cartridge, and the flow-through solution was combined with the sample in the same collection tube after centrifugation. Digested peptides were desalted using a SOLA SPE 96-well column (Thermo Fisher Scientific).

The LC-MS/MS analyses were performed on an Xcalibur 2.2 SP1 system coupled with a Q Exactive mass spectrometer (both Thermo Fisher Scientific). Peptide concentrations of all samples were adjusted to 20.6 ng/μl with 0.1% TFA. For each analysis, 10 μl of sample (corresponding to a total sample amount of 300 ng of proteins digested with trypsin) was injected. After injection, peptides were preconcentrated with 0.1% TFA on a trap column (AcclaimR PepMap 100 column, 100 μm i.d. by 2 cm, C18, 5-μm particle size, 100-Å pore size; Thermo Scientific) at a flow rate of 7 μl/min for 10 min. Subsequently, the analyte was transferred to the analytical column (AcclaimR PepMap RSLC column, 50 μm i.d. by 15 cm, C18, 2-μm particle size, 100-Å pore size; Thermo Scientific) and separated at 60°C using a 90-min gradient from 5 to 95% solvent B at a flow rate of 220 nl/min (solvent A: 0.1% formic acid; solvent B: 0.1% formic acid in acetonitrile). The gradient elution conditions were as follows: 0 to 55 min, 5% B to 30% B; 55 to 80 min, 30% B to 50% B; and 80 to 90 min, 50% B to 100% B. The mass spectrometer was operated in a data-dependent mode. Full-scan mass spectra were acquired at a mass resolution of 70,000 (mass range of 350 to 2000 *m/z*) in the Orbitrap analyzer. Tandem mass spectra of the 20 most abundant peaks were acquired in the linear ion trap by peptide fragmentation using collision-induced dissociation. Normalized collision energy was set to 27%, and an isolation width of 2.0 *m/z* was chosen.

Analyses of MS/MS spectra followed a similar protocol to that described for Di-Gly ubiquitinome analysis, with the following differences: Database searching was done using the *Arabidopsis* UniProt reference proteome with a 10-ppm mass tolerance for precursors. Relative protein quantification was performed using the MaxQuant software for label-free quantification analysis, and the search engine was Andromeda. Quantitation was carried out only for proteins with two or more unique peptide matches.

Quantitative proteomic analysis of isolated chloroplasts was performed at the Advanced Proteomics Facility, University of Oxford. This followed procedures similar to those described above for the analysis of whole-seedling samples, and for the analysis of Myc-tagged ubiquitin interactors.

### Annotation for subcellular and subplastidic localization and function

Proteins identified by LC-MS/MS were manually annotated with respect to subcellular and subplastidic localization and function. Information on protein characteristics, function, and subcellular and subplastidic localization was searched sequentially and manually in several public databases [The Arabidopsis Information Resource (*67*), UniProt (*65*), and National Center for Biotechnology Information (NCBI) (*68*)] and in the appropriate literature to annotate well-characterized chloroplast proteins. Two specialist databases, Plant Proteome Database (PPDB) (*20*) and AT_CHLORO (*21*), were used to assign chloroplast and subplastidic localizations for some less well-characterized proteins. For proteins not included in these databases, the TargetP prediction tool (*69*) was used to test for the presence of a transit peptide (a typical feature of chloroplast proteins), or the SUBA4 database (*70*) was used, which provides information on subcellular localization from published proteomic or fluorescent protein fusion datasets. Proteins associated with the chloroplast envelope were assessed by reference to a recently published proteomics study (*16*). Chloroplast proteins with unclear subplastidic localization were analyzed using the ARAMEMNON database (*71*) for the prediction of transmembrane domains to rule out the possibility of stroma or intermembrane space localization. Functions of proteins were classified with gene ontology (GO) terms using the Protein Analysis Through Evolutionary Relationship Classification System (*72*).

### RNA sequencing

Eight-day-old CDC48-DN and CDC48-WT plants were transferred onto MS agar medium supplemented with 4 μM estradiol and incubated for a further 2 days before whole seedlings were collected, weighed, and frozen in liquid N_2_. Three independent samples of each genotype were collected, and total RNA was extracted using TRIzol reagent (Invitrogen, Thermo Fisher Scientific). Sample RNA quality and integrity were assessed by agarose gel electrophoresis. RNA-seq and data analysis were performed by Shanghai HanYu Biotech laboratory.

For RNA-seq library preparation and sequencing, RNA was purified using Dynabeads Oligo(dT)_25_ (Invitrogen, Thermo Fisher Scientific) followed by deoxyribonuclease I (Takara) treatment. Library preparation used 100 ng of RNA per sample and was done using the NEBNext Ultra RNA Library Prep Kit for Illumina (NEB). After cluster generation on a cBot cluster generation system using the TruSeq PE Cluster Kit (Illumina), the prepared libraries were sequenced on Illumina Nova, resulting in paired-end reads.

To obtain clean reads, raw data (raw reads) in FASTQ format were processed using Trimmomatic v0.32 to remove data containing poly-N and low-quality reads. The clean reads were then mapped to the *Arabidopsis* reference genome (*67*) using bowtie2 v2.1.0. The expression levels of genes were calculated in FPKM (fragment per kilobase of gene per million reads mapped). Differentially expressed genes were identified using the P package DEGseq v1.20.0. Genes with log_2_ fold change greater than 1 or less than −1 and adjusted *P* value lower than 0.001 were classified as differentially expressed genes.

### Protein degradation assays

Protein degradation was investigated by using cycloheximide-chase degradation assays or lincomycin-chase degradation assays, as described previously (*73*, *74*) with modifications. Cycloheximide and lincomycin are translational inhibitors used to block the synthesis of nucleus-encoded proteins and chloroplast-encoded proteins, respectively.

To monitor degradation of PrfB3, wild-type protoplasts transfected with the *PrfB3-HA* construct were analyzed by cycloheximide treatment. Transformed protoplasts were incubated for 15 hours, and then, cycloheximide was applied to a final concentration of 10 μg/ml (dissolved in water), before further incubation for the durations indicated in the figure. The UPS dependency of PrfB3 turnover was determined by applying cycloheximide with and without bortezomib, and samples were analyzed after 6, 12, and 24 hours of incubation.

For lincomycin treatment, seedlings grown in vitro for 8 days were transferred to MS liquid medium (without sucrose) containing 400 μM lincomycin (dissolved in water) and then further grown under normal conditions for the durations indicated in the figure. Seeding samples (50-mg fresh weight) were flash-frozen in liquid N_2_ upon collection, and protein extracts were prepared as previously described (*51*). For the induction of the CDC48-DN and CDC48-WT constructs, 8-day-old transgenic plants were transferred to liquid MS medium containing 4 μM estradiol and incubated for 2 days; lincomycin was added during this incubation at 0, 12, 24, and 36 hours to achieve the desired periods of treatment. Experiments were performed with three biological replicates, and for each replicate, at least 10 seedlings were analyzed per genotype at each time point.

### In vivo retrotranslocation assays

In vivo retrotranslocation assays were performed as previously described with minor changes (*8*). First, FLAG-Ub was transiently overexpressed in 10^6^ protoplasts of each genotype (CDC48-DNa and CDC48-WTa) to increase detection sensitivity for higher molecular weight (ubiquitinated) forms of the chloroplast substrates (*7*). The transformed protoplasts were incubated for 15 hours, and then, bortezomib was applied to a final concentration of 5 μM before an additional 3-hour incubation. Subsequent fractionation steps to produce separate chloroplast and cytosol samples were all carried out on ice or at 4°C and used previously described procedures with modifications (*8*). Protoplasts were pelleted by centrifugation at 100*g* for 2 min and gently resuspended with protoplast-washing buffer [500 mM mannitol and 4 mM MES-KOH (pH 5.6)]. Then, the protoplasts were pelleted again, resuspended by gentle agitation in 500 μl of HS buffer [50 mM Hepes-NaOH (pH 8.0) and 0.3 M sorbitol] containing 0.5% PPIC and 5 μM bortezomib, and gently forced twice through 10-μm nylon mesh to release chloroplasts. The collected flow-through was centrifuged at 1000*g* for 5 min to produce a chloroplast-containing pellet and a cytosol-containing supernatant (S1). The pellet was gently resuspended in 500 μl of HS buffer, and the chloroplasts were purified using a two-step Percoll (Fisher Scientific) gradient (*45*). Intact chloroplasts were washed with 500 μl of HS buffer and then pelleted by centrifugation at 1000*g* for 5 min. The S1 sample was centrifuged at 10,000*g* for 15 min. The resulting supernatant (S10) was recovered and ultracentrifuged at 100,000*g* for 1 hour, producing a further supernatant (S100) that was concentrated to 50 μl by using Vivaspin 500 ultrafiltration spin columns; this was the cytosolic fraction. Subsequence detection of substrate ubiquitination in both chloroplast and cytosolic fractions was performed by immunoprecipitation (anti-FLAG) and immunoblotting as described above. Experiments were repeated three times, and similar results were obtained.

### Measurement of lipids and free FAs

Plant material was harvested as described above for quantitative proteomic analysis, except that for each genotype, six independent samples were processed. Measurement of lipids and FAs was performed by Shanghai Luming Biotechnology Co. Ltd. Lipids/FAs were extracted as described by Folch *et al.* (*75*) with modifications. Briefly, lipids/FAs were extracted from ~50 mg of frozen tissue powder using 1-ml chloroform:methanol (2:1, v/v), spiked with known amounts of isotope-labeled internal standards. The samples were vortexed for 30 s and then incubated at 4°C in an ultrasonic bath for 10 min. Subsequently, samples were placed at −20°C for 30 min, and in each case, the lower organic phase was recovered and centrifuged at 13,000*g* for 10 min at 4°C. The supernatants were collected, and the lipid extraction step was repeated. The cleared organic phases were combined and evaporated using a SpeedVac system. Last, samples were reconstituted in isopropanol:methanol (1:1, v/v).

Liquid chromatography was performed as described previously (*76*), with modifications, using an ExionLC System (Sciex) consisting of a binary high-pressure mixing gradient pump with degasser, a thermostated autosampler, and a column oven; the mass spectrometer was a QTRAP 6500+ (Sciex) equipped with an IonDrive Turbo V source. The temperature of the autosampler was set at 10°C. The mobile phases were acetonitrile:water (6:4, v/v) with 0.1% formic acid and 10 mM ammonium formate (eluent A) and acetonitrile:methanol (1:9, v/v) with 0.1% formic acid and 10 mM ammonium formate (eluent B). For each injection, a 5-μl sample was loaded into an Acquity UPLC HSS T3 column (2.1 mm i.d. by 10 cm, 1.7-μm particle size; Waters) using a 1.5-min initial flow of 0% B, a 3.5-min linear gradient from 0% B to 55% B, a 5-min linear gradient from 55% B to 60% B, a 3-min linear gradient from 60% B to 70% B, a 2-min linear gradient from 70% B to 90% B, and a 1-min linear gradient from 90% B to 100% B and held for 2 min. The flow rate was set at 350 μl/min. Last, the buffer was set back to 0% B, and the column was re-equilibrated for 2 min. Thus, the total running time was 20 min.

The MS method was performed in positive and negative electrospray ionization (ESI) modes, working in the time-scheduled multiple reaction monitoring (MRM) method. The source conditions were as follows: Curtain gas was 35 psi. Collision gas was medium. The ion spray voltage was −4500 V/+5500 V, and ion source gas 1 and ion source gas 2 were 40 and 45 psi, respectively. To evaluate the stability of the system during the whole analysis process, quality control samples obtained by mixing equal volumes (20 μl) of each sample were inserted into the analysis queue and determined three times in both positive and negative ESI modes. Spectrum data were collected using Analyst Software 1.7 (Sciex) according to the manufacturer’s instructions. Analysis of the spectra (alignment, peak picking, normalization, and peak integration) was performed using MRMPROBS (*77*). Lipids/FAs were identified by spectral and retention time matching with authentic compounds using an in-house custom library augmented with a library from Riken. Compounds were normalized relative to the internal standard.

### Statistical analysis

Statistical calculations (mean, SEM, *t* test) were performed using GraphPad Prism software. Statistical significance of differences between two experimental groups was assessed by using a two-tailed Student’s *t* test. Differences between two datasets were considered significant at *P* < 0.05.

## Notes

View/request a protocol for this paper from *Bio-protocol*.
