# Supplementary material for: Ubiquitin-based pathway acts inside chloroplasts to regulate photosynthesis
Source: Sci Adv. 2022 Nov 16;8(46):eabq7352. doi: 10.1126/sciadv.abq7352 (PMC9668298; doi:10.1126/sciadv.abq7352)
Supplement: Supplementary file 1 — Figs. S1 to S11 [file sciadv.abq7352_sm.pdf]

Science Advances
AAAS

Supplementary Materials for

Ubiquitin-based pathway acts inside chloroplasts to regulate photosynthesis

Yi Sun *et al.*

Corresponding author: Qihua Ling, qhling@cemps.ac.cn; R. Paul Jarvis, paul.jarvis@plants.ox.ac.uk

Sci. Adv. **8**, eabq7352 (2022)
DOI: 10.1126/sciadv.abq7352

The PDF file includes:

Legends for tables S1 to S10
Figs. S1 to S11

Other Supplementary Materials for this manuscript includes the following:

Tables S1 to S10

Supplementary Tables:

The following supplementary tables are provided separately.

Table S1. Identification of chloroplast proteins that co-immunoprecipitate with 6Myc-Ub.

Table S2. Identification of ubiquitinated proteins in wild-type chloroplasts using di-Gly ubiquitinomics.

Table S3. Identification of ubiquitinated chloroplast proteins in CDC48-DN plants using di-Gly ubiquitinomics.

Table S4. Chloroplast-encoded proteins with ubiquitination sites.

Table S5. Quantitative proteomic analysis of chloroplast proteins in CDC48-DN and CDC48-WT plants.

Table S6. Selected putative CHLORAD substrates identified by quantitative proteomics.

Table S7. Quantitative proteomic analysis of isolated CDC48-DN and CDC48-WT chloroplasts.

Table S8. Quantitative transcriptomic analysis of CDC48-DN and CDC48-WT plants.

Table S9. Lipidomic analysis of CDC48-DN and CDC48-WT plants.

Table S10. Primers used during the course of the study.

Supplementary Figures:

A

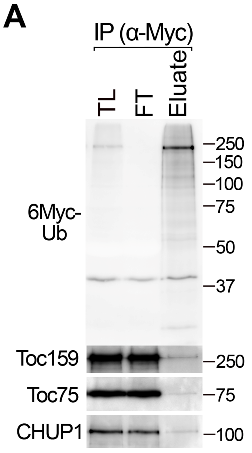

B

Protein Name	AGI number	Biological function	Chloroplast sublocalization	Ub Position
PrfB3	AT3G57190	Chloroplast gene expression	Stroma	113
Toc159	AT4G02510	Protein import	OEM	N/A
Toc33	AT1G02280	Protein import	OEM	N/A
Toc75	AT3G46740	Protein import	OEM	N/A
LOX2	AT3G45140	Lipid metabolism	IEM	N/A
RbcL	ATCG00490	Rubisco large chain	Stroma	N/A
PAO	AT3G44880	Chlorophyll metabolism	Stroma	N/A
PsaA	ATCG00350	Photosystem I subunit	Thylakoid	N/A
PsaB	ATCG00340	Photosystem I subunit	Thylakoid	N/A
PsbB	ATCG00680	Photosystem II subunit	Thylakoid	N/A
PsbC	ATCG00280	Photosystem II subunit	Thylakoid	N/A
PsbD	ATCG00270	Photosystem II subunit	Thylakoid	N/A
PetA	ATCG00540	Cytochrome b_6f subunit	Thylakoid	N/A
AtpA	ATCG00120	ATP synthase subunit	Thylakoid	N/A
AtpB	ATCG00480	ATP synthase subunit	Thylakoid	N/A

C

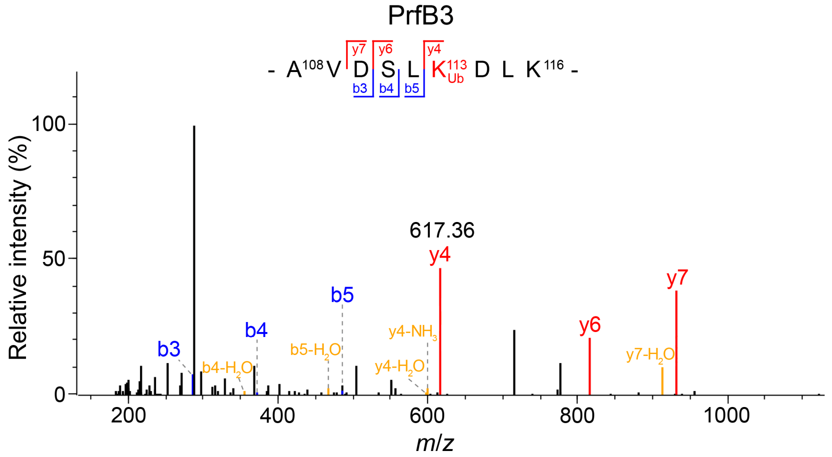

Fig. S1. **Identification of chloroplast ubiquitination by immunoprecipitation.**

(**A**) Detection of ubiquitinated proteins in chloroplasts purified from plants expressing Myc-tagged ubiquitin by immunoprecipitation and immunoblotting. Transgenic seedlings expressing ubiquitin with an N-terminal 6×Myc tag (6Myc-Ub) were used to isolate chloroplasts. The 6Myc-Ub chloroplasts were subjected to lysis and membrane solubilization prior to immunoprecipitation (IP) using anti-c-Myc affinity gel. Total lysate (TL; before immunoprecipitation was initiated), IP flow-through (FT), and IP eluate samples were analysed by immunoblotting using anti-Myc and the other indicated antibodies. Ubiquitinated proteins were enriched in the eluted samples, as indicated by the high molecular weight smears, compared with the input sample. (**B** and **C**) Identification of ubiquitinated chloroplast proteins by immunoprecipitation and mass spectrometry. The 6Myc-Ub elution sample shown in A was analysed by LC-MS/MS, and the data were analysed by MaxQuant. This identified six peptides for ubiquitin (covering 78.4% of the whole ubiquitin protein sequence), as well as the chloroplast proteins listed (B) (OEM, outer envelope membrane; IEM, inner envelope membrane). After trypsin digestion, a di-glycine remnant of ubiquitin remains covalently linked to target lysine residues of modified proteins (K-ε-GG), and this is identified by an increased mass of 114 kDa of the lysine residue. A ubiquitinated peptide was detected for the stromal protein PrfB3 (B,C), and the

associated fragmentation spectrum is shown (C). The y and b ions are shown in red and blue, respectively, while ions with H_2O or NH_3 shift are shown in orange. The b ions appear to extend from the N-terminus, and the y ions appear to extend from the C-terminus. The corresponding peptide sequence is presented, with the ubiquitinated K residue is marked in red. The *m/z* value of the corresponding fragment is indicated in black.

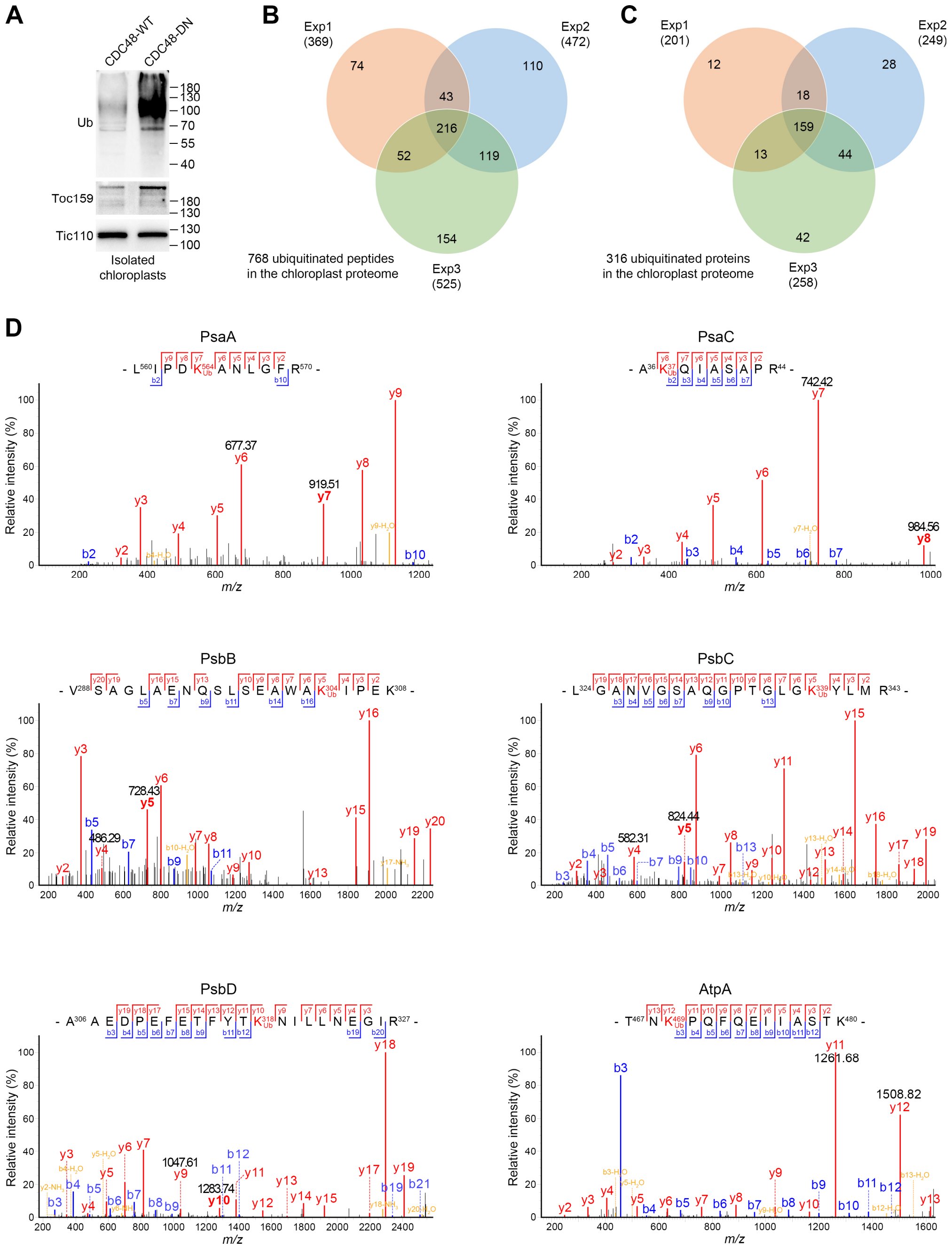

Fig. S2. Identification of ubiquitinated chloroplast proteins by di-Gly ubiquitinomics.

(**A**) Detection of the accumulation of ubiquitinated proteins in chloroplasts isolated from CDC48-DN plants by immunoblotting. Chloroplasts were isolated from CDC48-WT and CDC48-DN transgenic seedlings that had been induced with estradiol for two days. Induction of CDC48-DN was used to elevate the levels of ubiquitinated chloroplast proteins by blocking the CHLORAD system, in order to increase the chance of capturing ubiquitination events that would otherwise be below the level of detection, due to low stoichiometry or abundance. The purified chloroplasts were analysed by immunoblotting using anti-ubiquitin and the other indicated antibodies.

(**B** and **C**) Identification of ubiquitinated proteins and peptides in CDC48-DN chloroplasts by di-Gly ubiquitinomics. Proteins extracted from CDC48-DN chloroplasts like those shown in A were digested with trypsin and subjected to immunoprecipitation analysis using an anti-K-ε-GG antibody. The recovered ubiquitinated peptides were submitted to LC-MS/MS analysis, and the data were analysed by MaxQuant.

Three independent biological replicates were analysed (Exp1-3), and the overlaps among the identified ubiquitinated peptides (B) and proteins (C) are shown.

(**D**) Identification of ubiquitination sites in several chloroplast-encoded proteins by mass spectrometry. Many of the identified ubiquitinated proteins (B,C) were key photosynthetic complex components, and several of these are encoded by the chloroplast genome: PsaA, PsaC (PSI complex), PsbB, PsbC, PsbD (PSII complex), and AtpA (ATP synthase complex). Peptide sequences and representative spectra are shown for these proteins. The y and b ions are shown in red and blue, respectively, while ions with H_2O or NH_3 shift are shown in orange. The b ions appear to extend from the N-terminus, and the y ions appear to extend from the C-terminus. The ubiquitinated K residues are marked in red in the peptide sequences. The *m/z* values of the corresponding fragments are indicated in black.

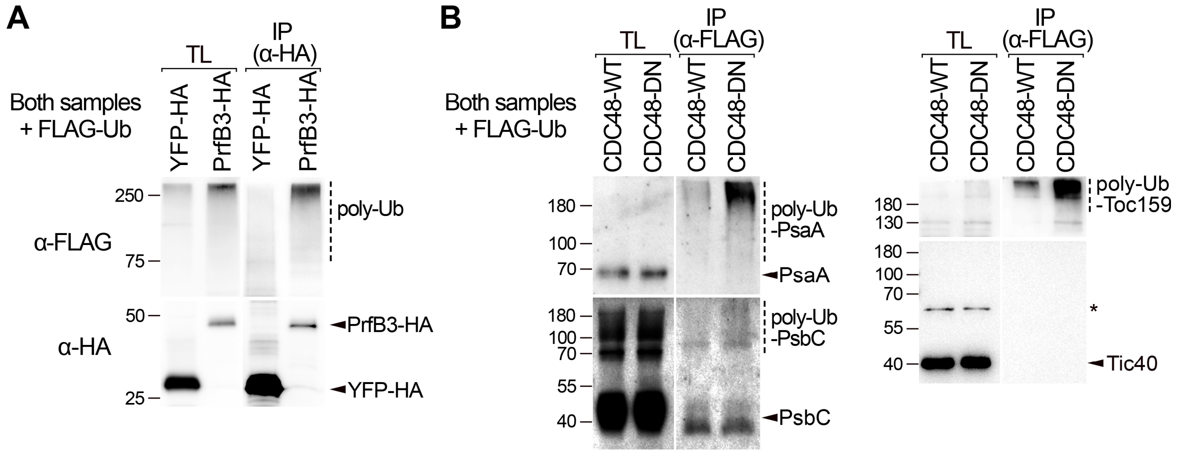

Fig. S3. Validation of the ubiquitination of proteins of the chloroplast interior.

(**A**) Validation of stromal protein PrfB3 using an in vivo ubiquitination assay. Wild-type protoplasts were cotransformed with constructs encoding HA-tagged PrfB3 (PrfB3-HA) and FLAG-tagged ubiquitin (FLAG-Ub), and the cells were subjected to immunoprecipitation (IP) analysis using anti-HA affinity gel. Total lysate (TL; before IP was initiated) and eluted IP samples were then analysed by immunoblotting using antibodies against: the FLAG tag, to detect polyubiquitinated forms (poly-Ub) of PrfB3-HA or YFP-HA; and the HA tag, to verify that the fusion proteins were present in the samples. Positions of molecular weight markers (sizes in kD) are shown to the left of the images.

(**B**) Validation of chloroplast-encoded proteins PsaA and PsbC using an in vivo ubiquitination assay. Protoplasts isolated from CDC48-WT and CDC48-DN transgenic plants were transfected with the FLAG-Ub construct and subjected to estradiol induction. The cells were then analysed by IP using anti-FLAG M2 affinity gel; in this case, ubiquitinated proteins were enriched instead of the target proteins, due to the difficulty of expressing tagged-chloroplast-encoded proteins in plants. Samples (TL, IP) were then analysed by immunoblotting using antibodies against the proteins indicated to the right of the images. Polyubiquitinated forms (poly-Ub) of PsaA and PsbC (and of the known CHLORAD substrate, Toc159) were detected in the IP samples, and these were distinctly larger than their unmodified forms in the TL samples. The poly-Ub forms also increased markedly in abundance in CDC48-DN, suggesting that they are normally subject to CHLORAD degradation. Tic40, which is not a CHLORAD substrate, acted as a negative control. The asterisk indicates a non-specific band. Molecular weight markers are indicated as in A.

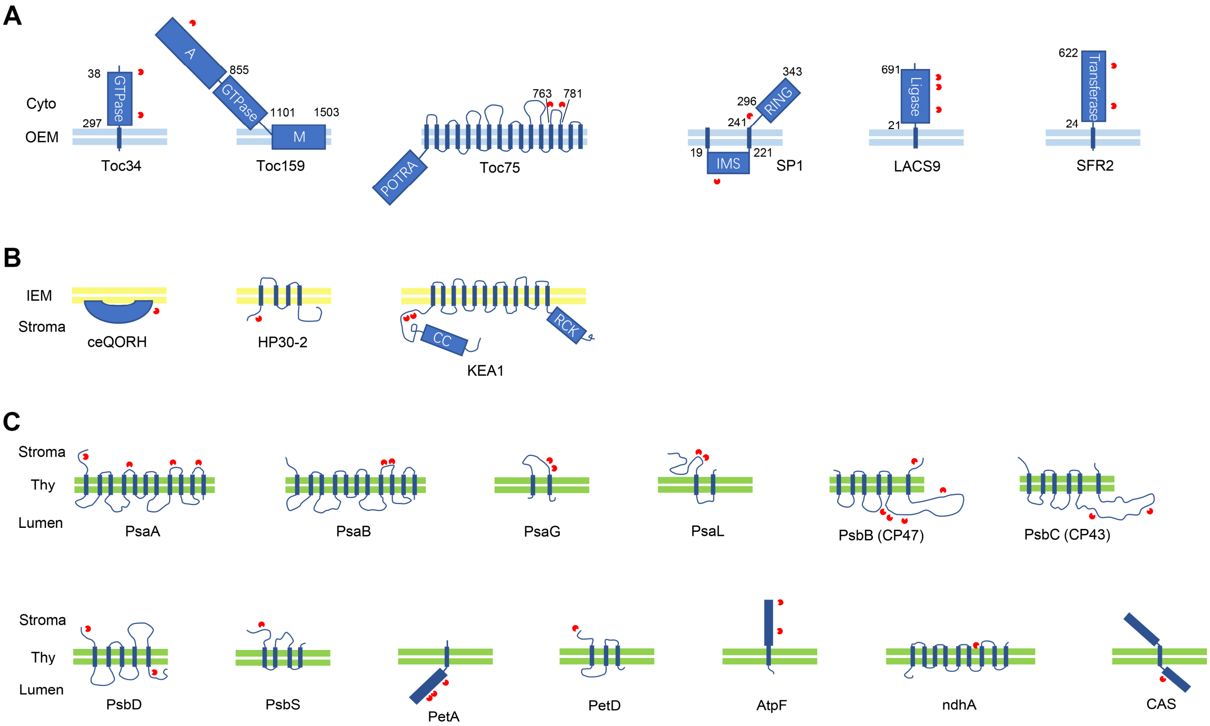

Fig. S4. Topological information on the ubiquitination sites of chloroplast proteins.

Schematic diagrams are shown for a representative selection of ubiquitinated chloroplast membrane proteins. The corresponding dataset is shown in table S3. The indicated proteins are resident in the outer envelope membrane (OEM; A), the inner envelope membrane (IEM; B), and the thylakoid membrane (Thy; C). Red symbols indicate the positions of ubiquitinated K residues. Numbers indicate the amino acid positions of protein domains; known domains are represented as dark blue shapes. Coloured double lines (light blue, yellow, green) indicate the relevant chloroplast membrane lipid bilayers. Cyto, cytosol. The noticeable lack of ubiquitination in the transmembrane domains implies that the ubiquitination of CHLORAD substrates occurs in situ and is domain specific; alternatively, it might reflect a technical difficulty with the identification of hydrophobic peptides by LC-MS/MS.

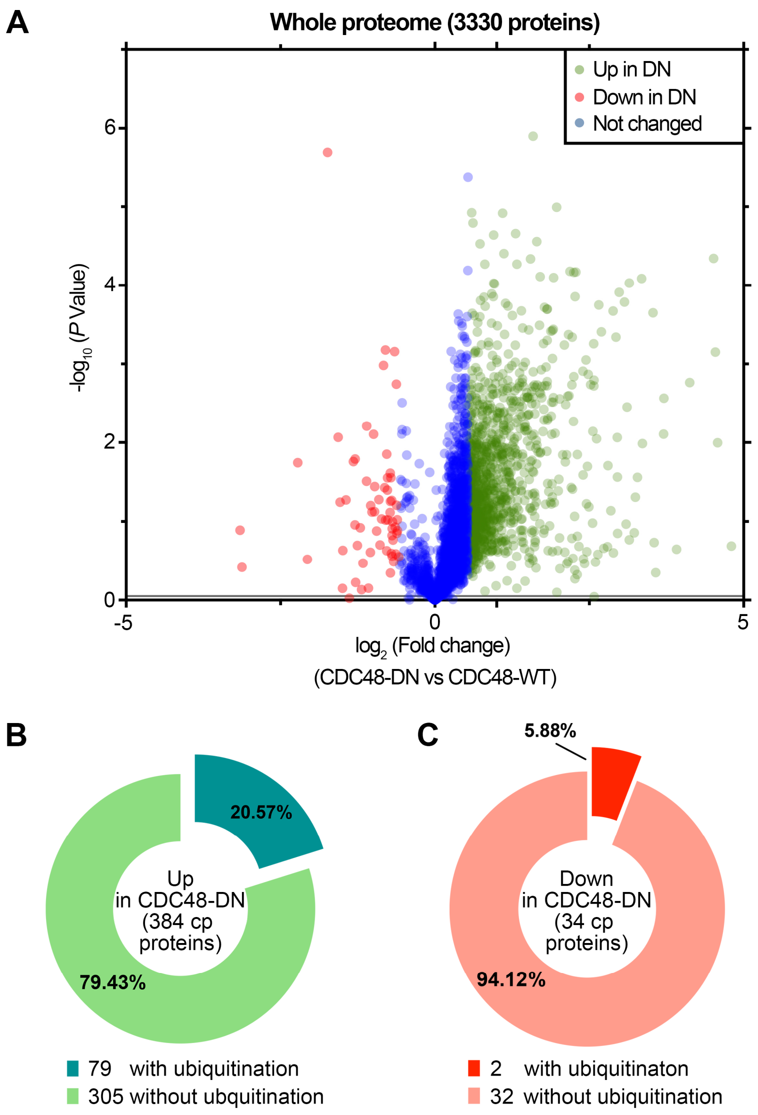

Fig. S5. Additional information on the quantitative proteomic analysis.

(**A**) Volcano plot representation of the data for all proteins identified in the quantitative proteomic analysis comparing CDC48-DN and CDC48-WT plants, before filtering for chloroplast-related proteins (Fig. 3). The graph shows $-\log_{10}$ *P* values plotted against $\log_2$ fold changes; proteins deemed to be showing a significant difference in abundance in CDC48-DN plants, relative to the CDC48-WT control, are indicated in green (Up) or red (Down). The corresponding dataset is show in table S5.

(**B** and **C**) Pie charts showing the proportion of chloroplast proteins identified in the quantitative proteomics analysis with at least one ubiquitination site. Chloroplast (cp) proteins over-accumulated in CDC48-DN (B), or reduced in CDC48-DN (C), were compared with the chloroplast ubiquitinome (Fig. 2), and in each case the percentage of proteins with detected ubiquitination is shown. The results show that proteins accumulated in CDC48-DN chloroplasts are more prone to be ubiquitinated, which suggests that chloroplast proteins are degraded by the UPS, via CDC48.

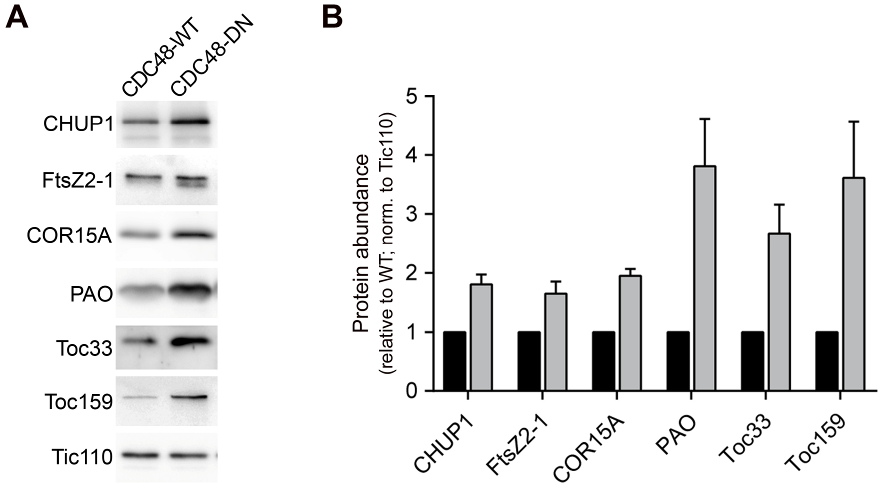

Fig. S6. Validation of the quantitative proteomics by using native protein antibodies.

Transgenic CDC48-WT and CDC48-DN plants were induced with estradiol for two days, and total protein extracts from these plants were analysed by immunoblotting. The antibodies used were against a selection of chloroplast proteins that showed over-accumulation in the quantitative proteomics analysis (Fig. 3); i.e., proteins deemed to be candidate CHLORAD substrates. The selection of proteins for analysis here was based in part on the availability of antibodies against the native protein. The proteins analysed also had different sub-organellar localizations, as follows: CHUP1 (OEM), FtsZ2-1 (IEM), COR15A, PAO (both stroma). Toc33 and Toc159, which are known CHLORAD substrates, were used as positive controls ; Tic110, which is not a CHLORAD substrate, was used as a sample normalization control. Typical immunoblotting results are shown (A). Band intensities for the candidate substrates were quantified and normalized to equivalent data for Tic110 (B). Data are means ± SEM from three biological replicates. The data show that the selected proteins specifically over-accumulate in CDC48-DN plants, verifying the proteomics data and suggesting that they are bona fide CHLORAD substrates.

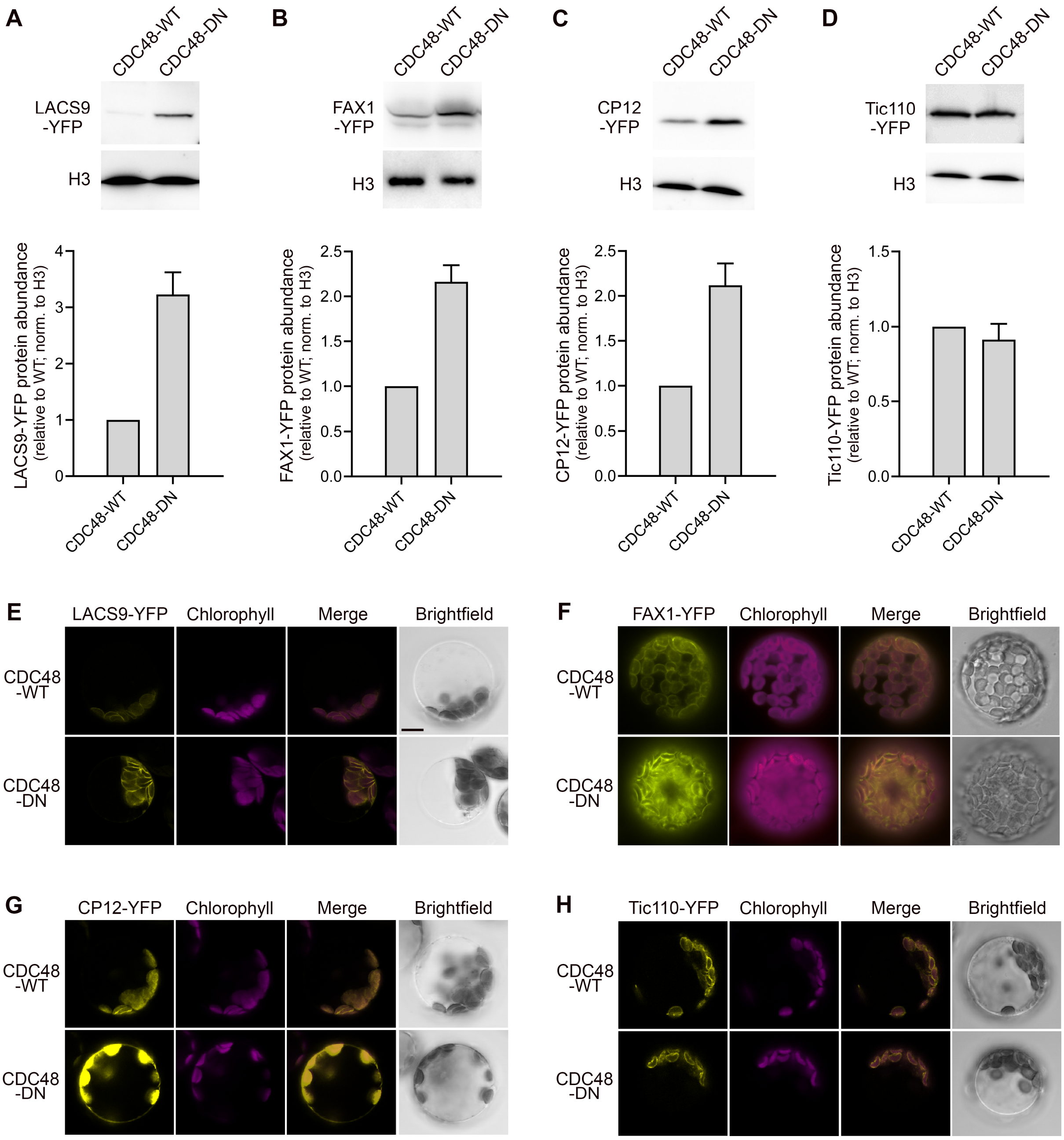

Fig. S7. Validation of the quantitative proteomics by analysing YFP-tagged proteins.

(**A** to **D**) Verifying the effect of CDC48-DN expression on candidate CHLORAD substrates by immunoblotting. For proteins that showed over-accumulation in the quantitative proteomics analysis (Fig. 3), but with no available antibody against the native protein, a tagging approach was employed for validation. Protoplasts from CDC48-WT and CDC48-DN transgenic plants were transiently transformed with constructs encoding fusion proteins with C-terminal YFP tags. The candidate substrate proteins analysed had different sub-organellar localizations, as follows: LACS9 (OEM; A), FAX1 (IEM; B), CP12 (stroma; C), Tic110 (control; D). The transfected protoplasts were incubated with estradiol for 15-18 h to induce CDC48 transgene expression, and then analysed by immunoblotting using antibodies against: the YFP tag, to detect the fusion proteins; and H3, as an internal sample normalization control. Typical immunoblotting results are shown (upper panels). Band intensities in the experiment shown, and in two additional similar experiments, were quantified; the values obtained for the YFP fusions were normalized using corresponding H3 values. Data are means ± SEM from three different experiments. The data show that the fusion proteins specifically

accumulate in CDC48-DN protoplasts, verifying the proteomics data and suggesting that the analysed candidates are bona fide CHLORAD substrates.

(**E** to **H**) Verifying the effect of CDC48-DN expression on candidate CHLORAD substrates by fluorescence microscopy. Protoplasts from CDC48-WT and CDC48-DN transgenic plants expressing the YFP-tagged proteins described in A-D were analysed by confocal microscopy. Chlorophyll autofluorescence was used to determine the localization of the YFP fluorescence signals relative to the chloroplasts. The LACS9-YFP (E), FAX1-YFP (F), and Tic110-YFP (H) signals all showed localization to the chloroplast envelopes, whereas the CP12-YFP signal (G) showed localization inside the chloroplasts; thus, all fusion proteins displayed the correct localization pattern. The intensity of the YFP signals for LACS9-YFP, FAX1-YFP and CP12-YFP increased markedly in CDC48-DN cells, implying that these proteins are degraded by CHLORAD at the chloroplasts. Tic110 is not a CHLORAD substrate, and so the intensity of the Tic110-YFP signal was unchanged in CDC48-DN cells. Brightfield images confirmed the intactness of the protoplasts. Scale bar, 10 µm. These data support a role for CHLORAD in degrading proteins resident in the chloroplast interior, rather than un-imported chloroplast preproteins that may exist in the cytosol.

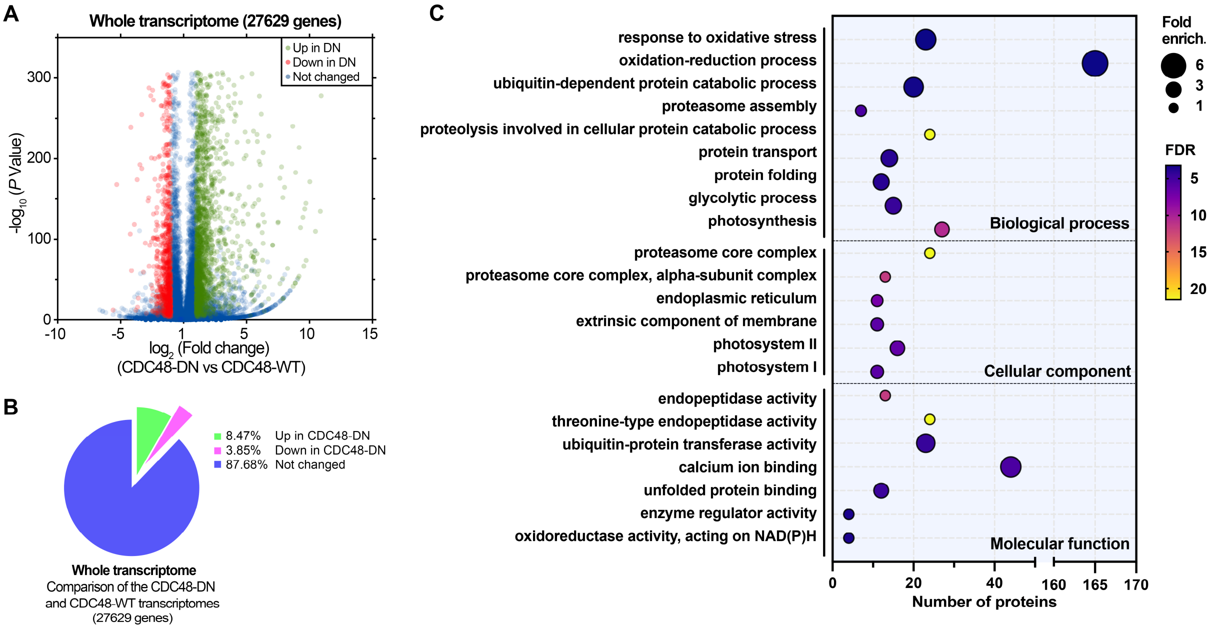

Fig. S8. Additional information on the RNA-seq transcriptomic analysis.

(**A**) Volcano plot representation of the data for all genes identified in quantitative transcriptomic analysis comparing CDC48-DN and CDC48-WT plants, before filtering for chloroplast-related genes (Fig. 3). The graph shows -$\log_{10}$ *P* values plotted against $\log_2$ fold changes; genes deemed to be showing a significant difference in expression in CDC48-DN plants, relative to the CDC48-WT control, are indicated in green (Up) or red (Down). The corresponding dataset is show in table S8.

(**B**) Pie chart showing the proportion of mRNAs in the whole transcriptome that are differentially expressed in CDC48-DN plants, as determined by the quantitative transcriptomic analysis. In general, more genes were up-regulated than down-regulated in response to CDC48-DN expression. In contrast, chloroplast-related genes showed the opposite trend, with more genes found to be down-regulated (Fig. 3D).

(**C**) Dot plot showing significantly overrepresented GO terms for mRNAs that are differentially expressed in CDC48-DN plants. Dot size indicates overrepresentation (fold enrichment) compared to the whole genome. Dot colour indicates False Discovery Rate (FDR; -$\log_{10}$ [*P* value]), where higher FDR values indicate more statistically significant enrichment. Dots are not shown for terms lacking statistically significant ($P < 0.05$) enrichment. In general, ubiquitin-dependent proteolytic processes are upregulated, whereas photosynthesis-related pathways are down-regulated.

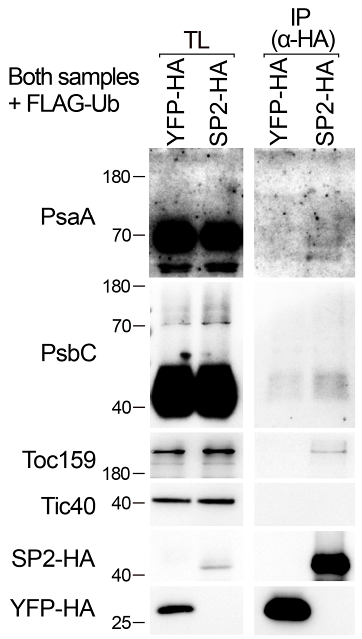

Fig. S9. Further analysis of the interaction between SP2 and putative CHLORAD substrates by co-immunoprecipitation.

The SP2-HA construct was used, together with a YFP-HA control construct, to co-transform Arabidopsis protoplasts. The cells were analysed by immunoprecipitation (IP) using anti-HA affinity beads. Total lysis (TL; before IP was initiated), and eluted IP samples were then analysed by immunoblotting using antibodies against: the HA tag, to verify the enrichment of SP2-HA and YFP-HA; PsaA and PsaC, to assess for interaction of SP2-HA with putative CHLORAD substrates; Toc159, to detect an established SP2-substrate interaction; and Tic40, to act as a negative control and confirm that the detected interactions are specific. Positions of molecular weight markers (sizes in kD) are shown to the left of the images.

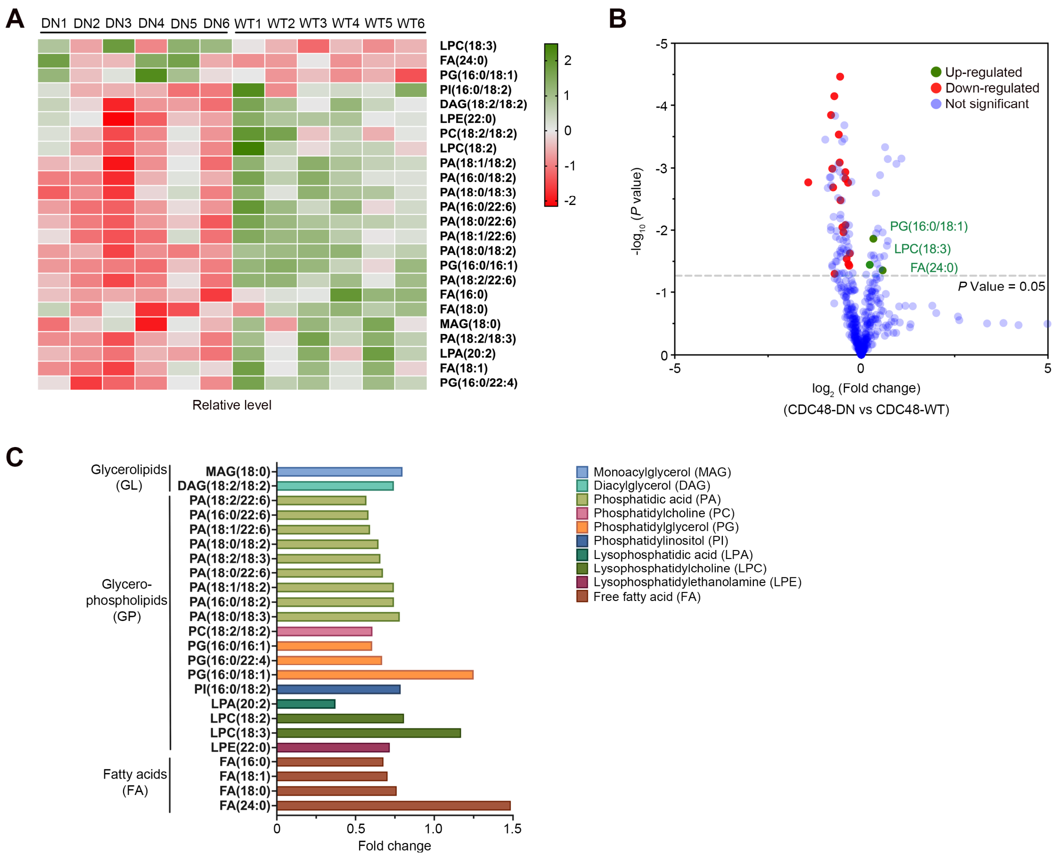

Fig. S10. Additional information on the fatty acid/lipid measurements.

(**A**) Heatmap showing the distinct FA and lipid profiles of CDC48-WT and CDC48-DN plants. Heatmap was applied to display the relative amounts of FAs and lipids, with a green colour gradient indicating high amounts and a red colour gradient indicating low amounts. Those FA and lipid species with significant changes in the CDC48-WT and CDC48-DN plant groups (six biological replicates for each group) were analysed in the heatmap (VIP [Variable Importance in Projection] > 1, *P* < 0.05). The numbers in the FA/lipid names describe the relevant FA chains; these numbers are presented in the format (number of carbons in the FA chain) : (number of double bonds in the FA chain).

(**B**) Volcano plot representation of the data for all FAs and lipids profiled. The graph shows $-\log_{10}$ *P* values plotted against $\log_2$ fold changes; species deemed to be showing a significant difference in abundance in CDC48-DN plants, relative to the CDC48-WT control, are indicated in green (Up) or red (Down) (VIP > 1, P < 0.05). The corresponding dataset is shown in table S9.

(**C**) Quantification of the changes in specific FA and lipid species in CDC48-DN. Bar graph showing relative amounts (fold change values) of species that were significantly changed in CDC48-DN plants, relative to CDC48-WT plants. The species shown are the same as those presented in A. Full names of the FA/lipid species are indicated to the right.

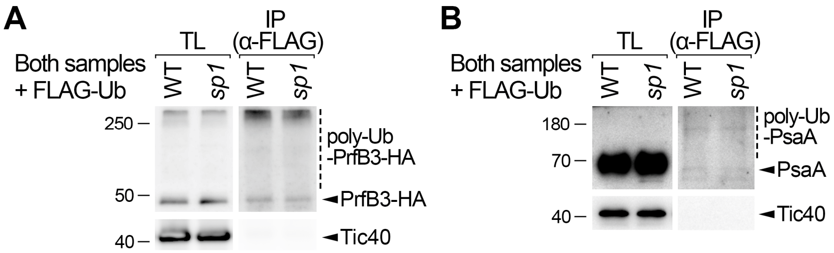

Fig. S11. Ubiquitination of internal CHLORAD substrates is not mediated by SP1.

(**A**) Constructs encoding PrfB3-HA and FLAG-tagged ubiquitin (FLAG-Ub) were used to co-transform protoplasts isolated from wild-type (WT) or *sp1-3* (*sp1*) mutant plants, and the cells were subjected to immunoprecipitation (IP) analysis using anti-FLAG M2 affinity gel to enrich ubiquitinated proteins. Total lysate (TL; before IP was initiated) and eluted IP samples were then analysed by immunoblotting using antibodies against: the HA tag, to detect unmodified PrfB3-HA and polyubiquitinated forms (poly-Ub) of PrfB3-HA, the latter being distinctly larger than the unmodified form; and Tic40, which is not a CHLORAD substrate and so acted as a negative control. Positions of molecular weight markers (sizes in kD) are shown to the left of the images. The abundance of the poly-Ub forms was not obviously different between WT and *sp1*, indicating that PrfB3 is not ubiquitinated by SP1.

(**B**) The FLAG-Ub construct was used to transform further WT and *sp1* protoplasts, and the cells were subjected to IP analysis using anti-FLAG M2 affinity gel to enrich ubiquitinated proteins. Samples (TL, IP) were then analysed by immunoblotting using antibodies against: PsaA, to detect unmodified PsaA and poly-Ub forms of PsaA, the latter being distinctly larger than the unmodified form; and Tic40, to act as a negative control. Molecular weight markers are indicated as in A. The abundance of the poly-Ub forms was not obviously different between WT and *sp1*, indicating that PsaA is not ubiquitinated by SP1.
