# Supplementary material for: Ubiquitin-based pathway acts inside chloroplasts to regulate photosynthesis
Source: Sci Adv. 2022 Nov 16;8(46):eabq7352. doi: 10.1126/sciadv.abq7352 (PMC9668298; doi:10.1126/sciadv.abq7352)
Supplement: Supplementary file 2 — Tables S1 to S10 [file sciadv.abq7352_tables_s1_to_s10.zip › sciadv.abq7352_table_s4.docx]

**Supplementary Table S4. Chloroplast-encoded proteins with ubiquitination sites.**

| **Gene name** | **AGI number** | **Ub no.^a^** | **Description** |
| --- | --- | --- | --- |
| PsaA | ATCG00350 | 4 | Photosystem I P700 chlorophyll a apoprotein A1 |
| PsaB | ATCG00340 | 2 | Photosystem I P700 chlorophyll a apoprotein A2 |
| PsaC | ATCG01060 | 4 | Photosystem I iron-sulfur centre protein |
| PsbB | ATCG00680 | 5 | Photosystem II CP47 reaction centre protein |
| PsbC | ATCG00280 | 2 | Photosystem II CP43 reaction centre protein |
| PsbD | ATCG00270 | 2 | Photosystem II D2 protein |
| PetA | ATCG00540 | 3 | Cytochrome f |
| PetD | ATCG00730 | 1 | Cytochrome b_6_-f complex subunit 4 |
| AtpA | ATCG00120 | 4 | ATP synthase subunit alpha, chloroplastic |
| AtpB | ATCG00480 | 8 | ATP synthase subunit beta, chloroplastic |
| AtpF | ATCG00130 | 2 | ATP synthase subunit b, chloroplastic |
| ndhA | ATCG01100 | 1 | NAD(P)H-quinone oxidoreductase subunit 1, chloroplastic |
| RbcL | ATCG00490 | 5 | Ribulose bisphosphate carboxylase large chain |

^a^Ub no., number of ubiquitination sites.

For the complete set of ubiquitinomics results, see Supplementary Table S3.
