# Supplementary material for: Ubiquitin-based pathway acts inside chloroplasts to regulate photosynthesis
Source: Sci Adv. 2022 Nov 16;8(46):eabq7352. doi: 10.1126/sciadv.abq7352 (PMC9668298; doi:10.1126/sciadv.abq7352)
Supplement: Supplementary file 2 — Tables S1 to S10 [file sciadv.abq7352_tables_s1_to_s10.zip › sciadv.abq7352_table_s6.docx]

**Supplementary Table S6. Selected putative CHLORAD substrates identified by quantitative proteomics.**

| **Gene Name** | **AGI number** | **Biological function** | **Ratio (DN/WT)** | **Chloroplast sublocalization** |
| --- | --- | --- | --- | --- |
| Toc159 | AT4G02510 | Protein import | 1.9 | OEM |
| Toc33 | AT1G02280 | Protein import | 1.7 | OEM |
| LACS9 | AT1G77590 | Fatty acid metabolism | 1.6 | OEM |
| OEP64 | AT3G17970 | Protein targeting | 1.8 | OEM |
| OEP24B | AT1G20816 | Metabolite transport | 1.5 | OEM |
| CHUP1 | AT3G25690 | Chloroplast relocation | 1.8 | OEM |
| FAX1 | AT3G57280 | Fatty acid metabolism | 2.1 | IEM |
| FtsZ2-1 | AT2G36250 | Chloroplast division | 1.5 | IEM |
| PAO | AT3G44880 | Chlorophyll metabolism | 2.1 | Stroma |
| CP12-2 | AT3G62410 | Photosynthesis | 90 | Stroma |
| COR15A | AT2G42540 | Chaperone | 1.6 | Stroma |
| Tic40 | AT1G06950 | Protein import | 1.3 | IEM |
| OEP80 | AT5G19620 | Protein targeting | 0.8 | OEM |

A selection of chloroplast proteins over-accumulated in CDC48-DN relative to CDC48-WT (fold change >1.5) is listed. Tic40 and OEP80 are not CHLORAD substrates, and thus serve as negative controls. OEM, outer envelope membrane; IEM, inner envelope membrane. For the complete set of quantitative proteomics results, see Supplementary Table S5.
