# Supplementary material for: Ubiquitin-based pathway acts inside chloroplasts to regulate photosynthesis
Source: Sci Adv. 2022 Nov 16;8(46):eabq7352. doi: 10.1126/sciadv.abq7352 (PMC9668298; doi:10.1126/sciadv.abq7352)
Supplement: Supplementary file 2 — Tables S1 to S10 [file sciadv.abq7352_tables_s1_to_s10.zip › sciadv.abq7352_table_s10.docx]

**Supplementary Table S10. Primers used during the course of the study.**

| **Primer name** | **Primer sequence (5’ to 3’)*** | **Used to generate …** |
| --- | --- | --- |
| CDC48-CDS-Fa | AAAAAGCAGGCTCCATGTCTACCCCAGCTGAAT | … *CDC48* CDS for plant transformation |
| CDC48-CDS-Ra | AGAAAGCTGGGTTATTGTAGAGATCATCATCGTCC |  |
| UBQ11-BamHI-F | AAGGATCCATGCAGATCT TTGTTAAGACTCTCACC | … *UBQ11* CDS for cloning into pE3n vector |
| UBQ11-NotI-R | AAGCGGCCGCTTAACCACCACGGAGCCTGAGG |  |
| LACS9-CDS-F | GGGGACAAGTTTGTACAAAAAAGCAGGCTCCATGATTCCTTATGCTGCTGG | … *LACS9* CDS for cloning into p2GWY7 vector |
| LACS9-CDS-ns-R | GGGGACCACTTTGTACAAGAAAGCTGGGTTGGCATATAACTTGGTGAGATC |  |
| FAX1-CDS-F | GGGGACAAGTTTGTACAAAAAAGCAGGCTCCATGGCTTCACAAATCTCTCAGC | ...  *FAX1* CDS for cloning into p2GWY7 vector |
| FAX1-CDS-ns-R | GGGGACCACTTTGTACAAGAAAGCTGGGTTGTATGAAGGACTAGTCGCAG |  |
| CP12-CDS-F | GGGGACAAGTTTGTACAAAAAAGCAGGCTCCATGGCAACTATAGCTACTGG | ...  *CP12* CDS for cloning into p2GWY7 vector |
| CP12-CDS-ns-R | GGGGACCACTTTGTACAAGAAAGCTGGGTTGTTGTCGTAAGTACGGCAC |  |
| PrfB3-CDS-F | GGGGACAAGTTTGTACAAAAAAGCAGGCTCCATGGCGGCAAAGATTATTGGT | ... *PrfB3* CDS for cloning into p2GW7 vector with C-terminal HA tag |
| PrfB3-CDS-ns-R | GGGGACCACTTTGTACAAGAAAGCTGGGTTAATCGCATCAATTGATCTTCTCA |  |
| SP2-CDS-F | GGGGACAAGTTTGTACAAAAAAGCAGGCTCCATGGGAGCTCAGAAGAGTATCCA | ... *SP2* CDS for cloning into p2GW7 vector with C-terminal HA tag |
| SP2-CDS-ns-R | GGGGACCACTTTGTACAAGAAAGCTGGGTTTGTTGATGAAGCAAGATTGGTG |  |
| attB1 | GGGGACAAGTTTGTACAAAAAGCAGGCT | ... complete Gateway recombination sites for cloning into pDONR201 |
| attB2 | GGGGACCACTTTGTACAAGAAAGCTGGGT |  |

*Nucleotides shown in red do not correspond to the target gene, but instead correspond to linker sequences.
